# A Single-Strand Annealing Protein Clamps DNA to Detect and Secure Homology

**DOI:** 10.1371/journal.pbio.1002213

**Published:** 2015-08-13

**Authors:** Marcel Ander, Sivaraman Subramaniam, Karim Fahmy, A. Francis Stewart, Erik Schäffer

**Affiliations:** 1 Nanomechanics Group, Biotechnology Center, TU Dresden, Dresden, Germany; 2 Department of Genomics, Biotechnology Center, TU Dresden, Dresden, Germany; 3 Division of Biophysics, Institute of Resource Ecology, Helmholtz-Zentrum Dresden-Rossendorf, Dresden, Germany; 4 Cellular Nanoscience, Center for Plant Molecular Biology (ZMBP), Universität Tübingen, Tübingen, Germany; Mount Sinai Hospital, CANADA

## Abstract

Repair of DNA breaks by single-strand annealing (SSA) is a major mechanism for the maintenance of genomic integrity. SSA is promoted by proteins (single-strand-annealing proteins [SSAPs]), such as eukaryotic RAD52 and λ phage Redβ. These proteins use a short single-stranded region to find sequence identity and initiate homologous recombination. However, it is unclear how SSAPs detect homology and catalyze annealing. Using single-molecule experiments, we provide evidence that homology is recognized by Redβ monomers that weakly hold single DNA strands together. Once annealing begins, dimerization of Redβ clamps the double-stranded region and nucleates nucleoprotein filament growth. In this manner, DNA clamping ensures and secures a successful detection for DNA sequence homology. The clamp is characterized by a structural change of Redβ and a remarkable stability against force up to 200 pN. Our findings not only present a detailed explanation for SSAP action but also identify the DNA clamp as a very stable, noncovalent, DNA–protein interaction.

## Abstract 2

During the annealing of single-stranded DNA molecules, homologous regions are first bound by monomers and subsequently secured by dimerization of single-strand annealing proteins, thereby triggering the growth of the nucleoprotein filament.

## Author Summary

High-fidelity repair of DNA breaks begins with accurate annealing of the complementary DNA strands. How sequence homology is efficiently detected—error-free—and eventually secured together on the molecular level is a fundamental, unsolved question. In this study, we show that the single-strand annealing protein Redβ initially recognizes sequence homology in the single DNA strands. Redβ then dimerizes to act as a molecular clamp of unprecedented strength that holds the strands together during the stage of strand annealing. This clamp secures and concludes the search for homology in the genome by initiating a nucleoprotein filament. Furthermore, the clamp may serve to hold broken chromosomes together against forces occurring during cell division. This mechanism may be a general paradigm for other protein recognition and nucleation processes.

## Introduction

In living cells, the genome is constantly being damaged by environmental agents [1]. Amongst an arsenal of repair pathways, double-strand break repair (DSBR), initiated by single-strand annealing proteins (SSAPs), is central for the maintenance of genomic integrity [2,3]. Recently, a new superfamily of SSAPs that includes RAD52 and Redβ from λ phage (GeneID: 3827055; protein accession: NP_040617) as prominent members has been identified [4,5]. In contrast to the RAD51/RecA family, which also binds single-stranded DNA (ssDNA) and initiates DSBR via single-strand invasion, these SSAPs are not ATPases and employ a mechanism that remains poorly understood [4,6]. In addition to a recently detected amino acid signature, SSAPs share several biochemical properties, including weak ssDNA but mostly no double-stranded DNA (dsDNA) binding and oligomerization to higher order, usually ring-like structures [7–10]. These spectacular structures have been the source of models relating the rings to the promotion of annealing [6,11]. However, ring structures have only been seen at high protein concentrations, which have also been reported to impair DNA binding and annealing activity [12–15]. Thus, it is unclear what role SSAP rings may play during annealing. Furthermore, in no case has the size of the reactant annealing complex or how homology detection proceeds and is eventually secured been determined. Consequently, the models based on the involvement of ring structures in homology detection and subsequent annealing catalysis [6,11] have been challenged [4,16]. In addition, several other key questions remain unanswered. How can homology be detected by proteins that bind DNA in a sequence-independent manner, and how is the fidelity of recombination ensured against false positives?

To address the above questions, we investigated the paradigm SSAP, Redβ from phage λ, using optical tweezers and three different single-molecule assays employing (i) cycles of dsDNA hairpin unzipping and annealing, (ii) preannealed Redβ filaments, and (iii) nicked dsDNA. These assays were complemented by gel shift, fluorescent correlation spectroscopy, and in vivo measurements to deduce that annealing promoted by Redβ is initiated by monomer, not ring, binding, which becomes stabilized upon a homology-dependent conformational change of a dimer that clamps the annealed strand with remarkable stability. We also support these conclusions by theoretical modeling of the annealing process.

## Results

### Redβ Weakly Holds DNA Together during Initial Strand Annealing

**Fig 1:**
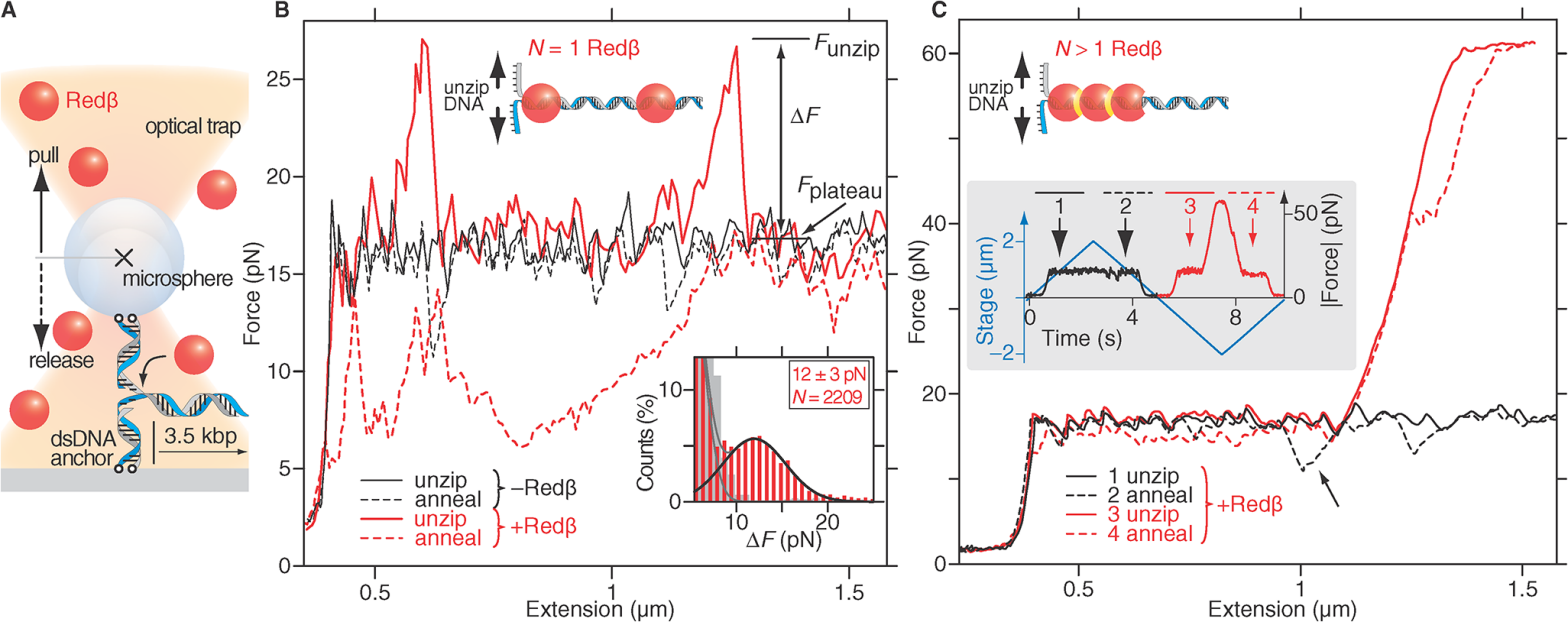
Optical tweezers separation force measurements reveal nucleoprotein–DNA interactions. (A) Schematic of optical tweezers unzipping experiments (not drawn to scale) using a 3.5-kbp DNA hairpin (blue-grey). Redβ monomers (red) do not bind dsDNA. (B) Typical traces of force versus DNA extension during DNA unzipping (solid lines) and zipping up/annealing (dashed lines) in the absence and presence of Redβ. The inset schematic illustrates two monomers that formed a nucleoprotein complex at random locations on the DNA hairpin after an unzipping/annealing cycle. Inset graph in (B): histogram of 2,209 rupture forces Δ*F* (red bars) relative to the plateau of regular unzipping. Low rupture forces are due to the background (gray bars in the absence of Redβ). A sum of Gaussians was fit to the histogram. The peak due to Redβ is indicated by a black line. (C) Force versus extension traces after repeated cycles of unzipping and annealing when unzipping became blocked (red curves). The arrow indicates the drop in annealing force that always occurred at the location and prior to the force increase in the subsequent unzipping cycle. The schematic indicates a nucleoprotein filament complex consisting of more than a Redβ monomer. Inset in (C): time course of the curves plotted in (C) showing the time sequence of annealing impairment and subsequent resistance. The initial time point of the rounds of unzipping traces was arbitrarily set to 0 s.

Redβ forms a nucleoprotein filament whilst annealing DNA [12,17]. To investigate the mechanism for DNA strand annealing by SSAPs at single-molecule resolution, we used optical tweezers [18–22]. Optical tweezers are a sensitive position and force transducer, whereby a small particle—typically a microsphere used as a handle for the experiment—is held in a tightly focused laser. We designed a dynamic optical tweezers experiment to repeatedly unzip/zip up a DNA hairpin tethered between a microsphere and a surface in the presence and absence of Redβ (Fig 1A and 1B; Section A, Fig AA, and Table A in S1 Text). To unzip/zip up the DNA hairpin, the surface was moved back and forth relative to the stationary trapping laser using a piezoelectric translation stage (blue lines in inset of Fig 1C) and recorded the position and force on the DNA-tethered microsphere as a function of time. From the position relative to the DNA anchor point, we calculated the DNA extension and plotted the force versus this extension (Fig 1B). In a typical experiment lasting multiple rounds of unzipping and annealing, we observed the following events: Redβ had no effect on the initial unzipping force (not shown), concordant with its inability to bind dsDNA. However, upon force release, DNA strand annealing during zipping up was impaired in the presence of Redβ, indicating Redβ binding to the exposed single DNA strands without the promotion of DNA annealing. The impairment showed up as a pronounced hysteresis and drop in force compared to strand annealing without Redβ (Fig 1B). Subsequent unzipping in the presence of Redβ exhibited distinct force peaks above the control unzipping plateau (control *F*_plateau_ ≈ 17 pN; Redβ force peaks Δ*F = F*_unzip_−*F*_plateau_ = 12 ± 3 pN, where *F*_unzip_ is the maximum rupture force at which the dsDNA unzips; Fig 1B and inset). These 12-pN force peaks were typically observed at high loading rates (≈800 pN/s) and occurred at the same position as the annealing hysteresis, indicating the persistence of Redβ binding. At low loading rates (≈40 pN/s), the annealing hysteresis and unzip force peaks were not apparent, implying that the binding interaction was weak [23]. Notably, the number of force peaks accumulated with increased number of unzip/zip up cycles, again suggesting that Redβ remains attached after DNA annealing. After repeated unzipping cycles, we observed a dramatic transition of dsDNA to an extremely stable state. Within one unzipping cycle (inset Fig 1C), resistance increased up to a plateau at ≈60 pN, which corresponds to the known force for the overstretching transition of dsDNA (here, of the dsDNA anchor segments, Fig 1C, [24]). Finally, after the 60-pN plateau, the DNA ripped off its anchors (not shown). As noted above for the 12-pN force peaks, immediately prior to these events, a drop in force during annealing was observed at the same position as the subsequent development of >60 pN resistance (Fig 1C arrow and inset).

### High Forces Are Required to Dissociate Nucleoprotein Filaments

**Fig 2:**
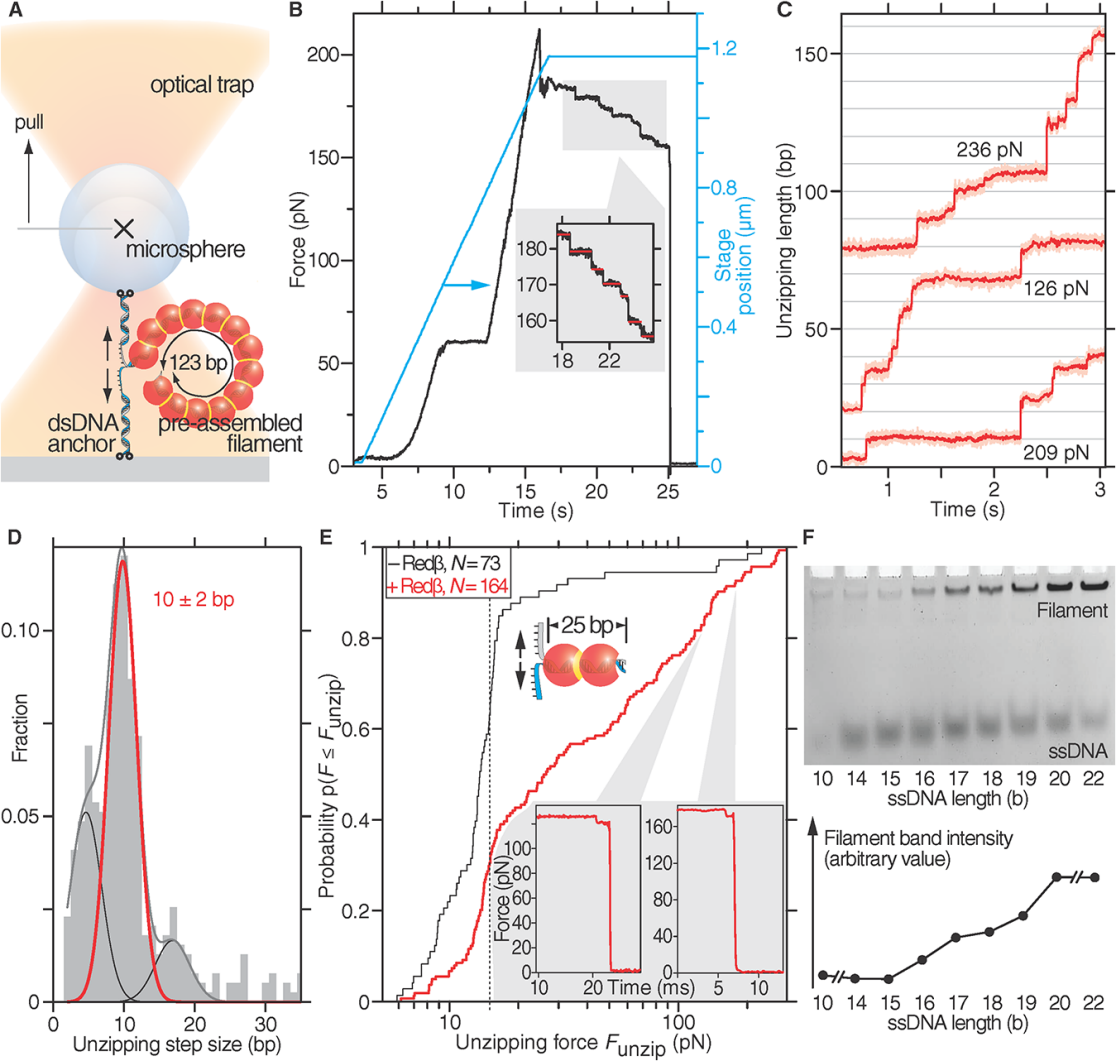
Nucleoprotein filaments consisting of at least a dimer resist substantial force. (A) Schematic of high-force optical tweezers experiments to unzip preassembled nucleoprotein filaments (not drawn to scale). (B) A 123-bp nucleoprotein filament was subjected to increasing load forces (black line, left-hand axis) by moving the stage laterally (cyan line, right-hand axis). The inset shows a magnification of the stepwise dissociation after the stage movement was stopped. The horizontal red lines indicate steps. (C) Unzipping length as a function of time during the rupture of a 123-bp Redβ filament under constant force. Three typical traces are offset for clarity. (D) Unzipping step-size histogram of 391 Redβ dissociation events from (C). The step size was fitted as the sum of Gaussian curves (grey line) with the main peak (red line) occurring at 10 ± 2 bp and side peaks (black line) at about 5 bp and 17 bp. (E) Unzipping forces *F*_unzip_ of a 25-bp region, preassembled with Redβ (red line, schematic) and without Redβ (black line), are plotted as a cumulative distribution function. The vertical dashed line indicates the average DNA unzipping force *F*_unzip_ without Redβ. The insets show two examples of the stepwise drop in force prior to rupture taken from the indicated places. (F) Electrophoretic mobility shift assay to measure the minimum length required for a stable Redβ nucleoprotein filament. Complementary oligonucleotides of increasing lengths as indicated were incubated with a molar equivalent of Redβ before electrophoresis. The mean band intensities of the filament bands are plotted underneath the gel image.

Two unexpected observations were made in the experiments summarized in Fig 1. First, Redβ apparently interfered with DNA annealing in the first rounds of unzipping. Second, after this interference, which presumably reflects stochastic Redβ binding, nodes resistant to unzipping accumulated, first to scattered ≈12-pN sites and then dramatically to >60-pN resistant segments. To understand these observations, we evaluated the stable Redβ nucleoprotein filament directly by preassembling a 123-bp filament (Fig 2). To exert forces large enough to rupture the filament, we had to stabilize the anchors (Section A, Fig AB, and Table A in S1 Text) and optimize the optical trap for high and constant forces. While applying an increasing force to the filament, we first observed the overstretching transition of the dsDNA anchor strands at ≈60 pN (Fig 2B, compare with Fig 1C). Subsequently, the nucleoprotein filament dissociated in a stepwise manner, with unzipping forces exceeding 200 pN (Fig 2B and inset)—much larger than the force peaks observed in the dynamic hairpin unzipping assay. Since we strained the dsDNA anchors several seconds before reaching these high forces, we expect that the dsDNA anchor strands were fully unwound [25] and that the disassembly steps were due to nucleoprotein filament disassembly and not due to internal DNA transitions [26]. The stepwise disassembly suggested that Redβ molecules dissociated individually from the nucleoprotein filament. We cannot deduce whether the protein dissociated completely from the DNA or was still bound to one of the strands. Since the force and extension in this measurement changed with every step, it was difficult to determine the length of dissociated DNA per step. To measure this length and, thus, the number of bases per Redβ disassembly step, we used a three-dimensional force feedback operating at a constant force of ≈200 pN (Fig 2C) and an unbiased step finding algorithm [21]. After converting the observed step sizes from nanometers to base pairs accounting for the extension of the DNA, the main peak has an average unzipping step size of 10 ± 2 bp (Fig 2D). This size agrees well with atomic force microscopy measurements of 11 ± 3 bp bound by each Redβ monomer within the nucleoprotein filament [4] and supports the conclusion that individual Redβ molecules were disassembled from the filament. The other peaks may be due to partial or dimer dissociation or possibly occasional slow transitions of the dsDNA anchor strands from stretched DNA (S-DNA) to peeled DNA, which should not affect the main peak [26]. As well as the stepwise disassembly, the data show that the nucleoprotein filament is extraordinarily stable against strand unzipping forces and indicate that (i) the >60-pN force plateau observed in Fig 1 arose from a stable Redβ filament and (ii) the 12-pN force peaks did not arise from a stable Redβ filament but rather from a different binding event, likely to be a precursor to stable filament assembly.

The 123-bp Redβ filament should include ≈12 Redβ monomers (i.e., 123/10). To evaluate the stability of a presumptive dimer filament, we set up the same pulling assay using Redβ—annealed, 25-nucleotide, complementary ssDNAs (Fig 2E, Fig AC and Table A in S1 Text). For more than 20% of the traces, we measured forces larger than 100 pN with maximum forces also exceeding 200 pN. Moreover, the separation occasionally (in 15 out of 164 traces) occurred in a stepwise manner (insets Fig 2E), again indicating the disassembly of individual Redβ monomers from the short nucleoprotein filament. Furthermore, for 50% of the recorded curves, forces greater than the 12-pN force peaks observed in Fig 1 were recorded, with an average of more than 30 pN (*F*_unzip_ = *F*_plateau_ + Δ*F* ≈ 17 pN + 12 pN ≈ 30 pN). In contrast, only 10% of annealed strands without Redβ exceeded this value (Fig 2E). The broad unzipping force distribution is probably implicit to the force-induced dissociation process [23]. Because the short 25-bp filament consisted most likely of a Redβ dimer and separation forces were much larger than the initial 12-pN force peaks recorded when unzipping hairpins, these data suggest that (i) the 12-pN force peaks were caused by random monomer binding events, (ii) dimer formation is the decisive step in strongly clamping strands together, (iii) a lateral contact between Redβ molecules has formed upon dimerization, and (iv) a dimer or trimer Redβ filament can form a stable complex sustaining forces of up to 200 pN.

To evaluate dimer formation in nucleoprotein filament formation, we used an electrophoretic mobility shift assay (EMSA). In the assay, we annealed complementary ssDNA oligonucleotides of different lengths from 10 to 22 b in the presence of a fixed concentration of Redβ at a Redβ/ssDNA molar ratio ≤2. Nucleoprotein filament formation required a minimum length of 16 bp and saturated at 20 to 22 bp (Fig 2F), again indicating that at least two Redβ molecules must be bound to establish a stable complex.

### Redβ Forms Strong Lateral Contacts

**Fig 3:**
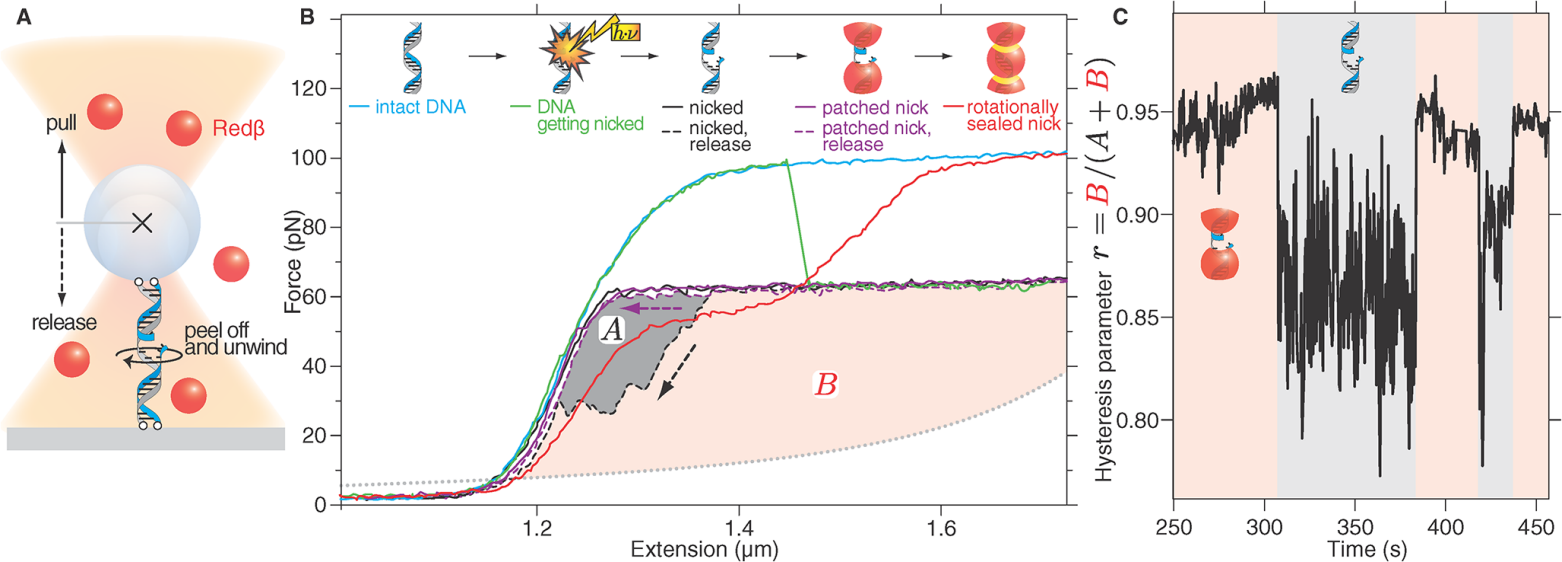
Lateral interaction of Redβ monomers reinforces the filament. (A) Schematic of optical tweezers DNA stretching experiments. (B) Force-extension plot of dsDNA attached to the microsphere and surface by all four strand termini in the presence of Redβ. Intact DNA shows a high-force plateau typical for rotationally constrained dsDNA (cyan line). A nick introduces rotational freedom leading to a plateau at ≈60 pN (green line) and hysteresis during release (black dashed line, grey area A). Subsequently, the hysteresis disappeared (purple dashed line), and repeated cycles plateaued at 60 pN (purple solid line). Occasionally, the higher-force plateau was reattained (red line). In these cases, the force-extension curve suggests that the dsDNA was sealed by Redβ but underwound. (C) Time course of the hysteresis parameter *r*. The hysteresis parameter *r* is the ratio between the red area (B) and the total area (A + B) demarcated in (B) by the measured force-extension curves and the expected force-extension curve of ssDNA (dotted line in [B]). The nicked, hysteresis state is characterized by $\bar{r}=0.86\pm 0.04$ (mean ± standard deviation) versus $\bar{r}=0.95\pm 0.01$ for the patched state. Each stretch/release cycle was 1-s long and produced one point in the time course of *r*.

In a third optical tweezers application, we employed a DNA stretching assay with all four DNA ends attached to the microsphere and the surface, thereby imposing a rotational constraint onto the dsDNA (Fig 3A). Without this constraint (i.e., with anchor points consisting of a single attached strand), the torque that the helical dsDNA experiences upon stretching is released by a free rotation of the DNA around its anchors. Because of this rotation, the DNA overstretches at the well-known value of ≈60 pN [24,27]. The overstretching plateau in our experiment at 100 pN and the absence of hysteresis upon return verified the torsional constraint imposed by the four anchored termini (Fig 3B, cyan line) [28, 29]. Occasionally, one of the strands incurred a nick, likely due to laser-induced photo damage. A nick introduces rotational freedom to the DNA and showed up as a sudden drop of the force plateau (Fig 3B, green line) to ≈60 pN and the occurrence of hysteresis (Fig 3B, black solid and dashed lines; Section B and Fig B in S1 Text; [30]). The hysteresis is caused by the nicked strand, which has peeled off from the other load-bearing strand [30]. Reannealing of the peeled-off strand requires additional time leading to hysteretic behavior. The presence of Redβ had no detectable influence on the level of the force plateaus. However, it suppressed the hysteresis through many cycles of stretching and reannealing (Fig 3B, purple solid and dashed line, Fig 3C). This suppression indicates that Redβ patched the nick and prevented single strands from peeling off at both the 3′ and 5′ ends of the nicked strand while allowing free rotation of the nicked strand around the continuous strand (Fig 3B). Occasionally, the 100-pN force plateau was reattained (red solid line in Fig 3B). In these cases, the path of the force-extension curve suggests that the DNA was fully sealed but partially unwound [31]. Notably, dsDNA in the Redβ nucleoprotein filaments also appears to be underwound [4]. In accord with the ssDNA binding data, the single-molecule stretching data is consistent with Redβ polymerization sealing the nick and constraining rotational freedom. In addition to clamping the DNA strands together, this constraint and the sustained loads imply that neighboring Redβ molecules form a stable lateral contact; otherwise, a filament would be free to rotate in this assay.

### Redβ Changes Its Structure upon Annealing

**Fig 4:**
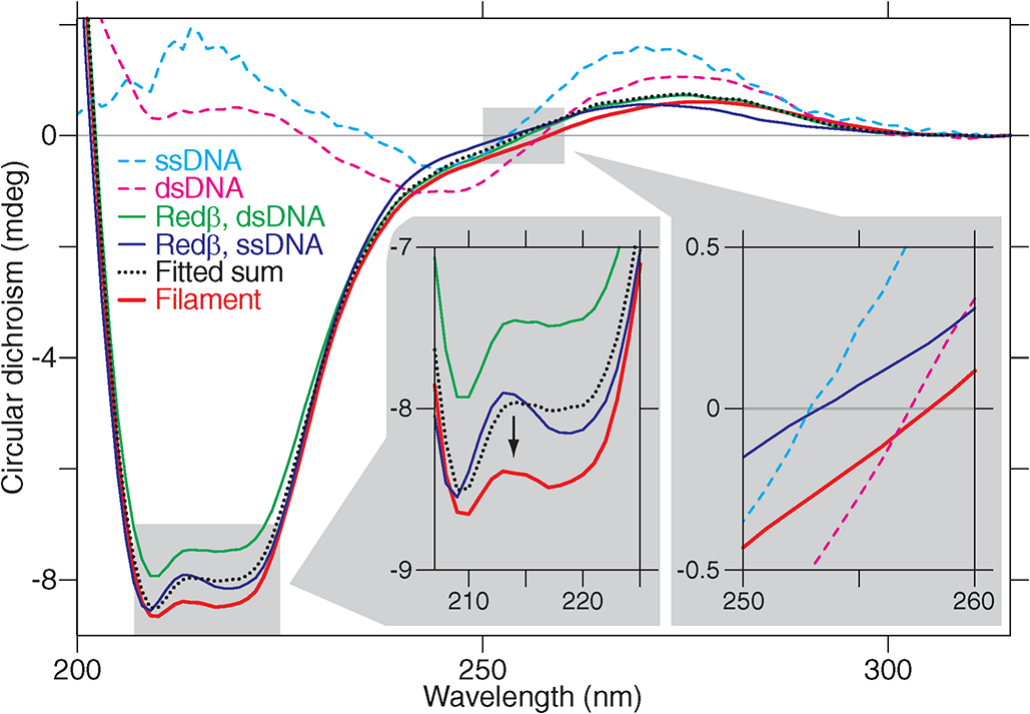
Circular dichroism (CD) suggests a structural change of Redβ upon filament formation. Circular dichroism spectra show a decrease for the nucleoprotein filament (left inset shows a magnified view). The dotted black line is a fit of arbitrary contributions of the spectra of ssDNA, dsDNA, free Redβ, Redβ +ssDNA, and Redβ +dsDNA such that the concurrently recorded absorbance spectra have a maximal overlap with that of the filament (see Fig C in S1 Text for details). In this manner, the decreased circular dichroism cannot be accounted for and, therefore, must stem from a structural change. The right inset shows a bathochromic circular dichroism shift between ssDNA, dsDNA, and filament.

The single-molecule observations correlate with the two known modes of Redβ DNA binding—that is, loose ssDNA binding and stable filaments upon annealing two complementary strands. However, the remarkable strength of the Redβ-annealed dsDNA filaments was unexpected. At >200 pN, the filaments are amongst the strongest protein interactions yet described, which we term “DNA clamping.” The two DNA binding modes and particularly the onset of DNA clamping suggests that Redβ undergoes a conformational change. To examine this proposition, we employed circular dichroism (CD) and observed a shift between ≈210–220 nm, indicative of a structural change of Redβ after annealing and filament formation (Fig 4, left inset, Section C and Fig C in S1 Text, [32]). In addition to the structural change of Redβ, the zero crossing of the filament trace (Fig 4, right inset) was near that of dsDNA suggesting the nucleoprotein filament contains underwound dsDNA close to B-form DNA. Thus, a structural change, likely upon dimerization, accompanies nucleoprotein filament growth and correlates with Redβ DNA clamping.

### In *Escherichia coli*, the Redβ Concentration for Recombineering Is Less Than 150 nM

**Fig 5:**
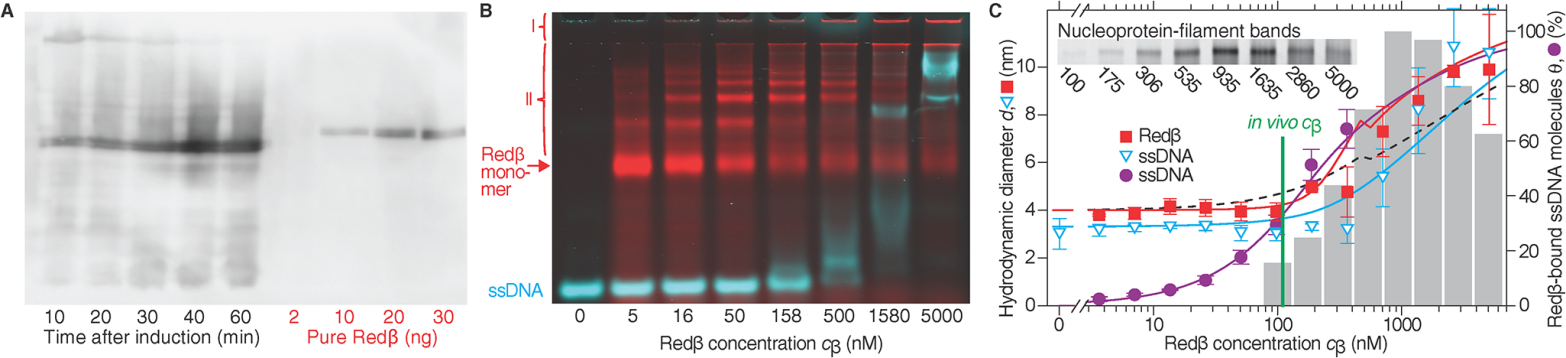
Redβ oligomerizes with increasing concentration. (A) Western blot after gel electrophoresis under denaturing conditions to determine the in vivo concentration of Redβ in *E*. *coli* induced to express at optimal conditions for Redβ-mediated homologous recombination. The band intensities were quantified and compared with purified StrepII-tagged Redβ (slightly slower migration due to the StrepII tag) loaded at the indicated concentrations. (B) Gel electrophoresis under nondenaturing conditions. Redβ banding pattern (red bands) indicates a concentration-dependent multimerization, which is not due to binding of ssDNA (cyan; 30 b, *c*_ssDNA_ = 1 nM; see also Fig D in S1 Text). Below the gel pockets and stacking gel (I), bands due to oligomers and rings (II) are visible. (C) Hydrodynamic diameter (left axis) of Redβ (red squares), ssDNA (open cyan triangles), and their cross correlation (right axis, purple circles) based on fluorescence correlation spectroscopy (FCS) as a function of Redβ concentration *c*_*β*_ averaged over three experiments. Error bars denote standard deviation. All plotted lines are calculated using our annealing model to compare the fit to the corresponding data points. The solid red line is based on the assumption of isodesmic growth with nucleation of a dimer (*a*_dimer_ < *a*, reduced chi-squared value $\chi_{\text{r}ed}^{2}=2.4$), the dashed black line assumes isodesmic growth without nucleation (*a*_dimer_ = *a*, $\chi_{\text{r}ed}^{2}=6.3$). The intensities of the inset nucleoprotein-filament gel bands obtained under nondenaturing conditions are shown as the grey histogram. The green line indicates the mean in vivo Redβ concentration of 110 nM.

The EMSA data (Fig 2F; [4]) supported by the single-molecule step-size measurements in Fig 2, indicate that stable Redβ nucleoprotein filament formation initiates when two adjacent Redβ molecules promote annealing of complementary DNA strands. In contrast, the spectacular multimeric rings formed by Redβ and RAD52 in the absence of DNA in vitro have been proposed to be the initiating agents [6,11,15,17,33]. For Redβ, ring formation in vitro requires protein concentrations above 0.8 μM. To find out whether rings are important in vivo, we measured the Redβ concentration at empirically determined optimal recombineering conditions (Fig 5A) [34–37]. In four biological repeats, the Redβ concentration in *E*. *coli* did not exceed 150 nM, corresponding to less than 350 molecules of Redβ per *E*. *coli* cell [38], which is well below the in vitro concentration required for ring formation [4,9].

### At In Vivo Concentrations, Redβ Is Mostly in Its Monomeric Form

To determine the size of the reactant Redβ–ssDNA complex as a function of the Redβ concentration (*c*_*β*_), we used gel electrophoresis assays and single-molecule fluorescence measurements (Fig 5B and 5C, Section D, Fig D, and Table B in S1 Text). In the presence (Fig 5B) and absence (Fig D in S1 Text) of ssDNA, Redβ showed regular band patterns indicating self-association to form oligomers with increasing oligomerization at increasing concentration (Section D in S1 Text). The broad mobility shifts of ssDNA at 158 nM and above in Fig 5B concord with the known weak binding by Redβ [39], and we attribute the smearing to the release of Redβ during electrophoresis. Controls verified that Redβ did not interact with dsDNA under these conditions (not shown).

Formation of the nucleoprotein filament was evaluated by gel electrophoresis after incubation of two complementary ssDNA oligonucleotides (30-nt and 50-nt long) with increasing concentrations of Redβ. The filament was first observed at 100 nM and increased to a maximum at *c*_*β*_ ≈ 1 to 2 μM, and it subsequently decreased (Fig 5C, inset gel, band intensities quantified in the grey histogram). Because of the limited sensitivity of the gel assay, we used tagged molecules for single-molecule fluorescence measurements employing fluorescence correlation spectroscopy (FCS, [40,41]). The average hydrodynamic diameter of both Redβ and ssDNA was measured as a function of *c*_*β*_ (red squares and cyan triangles in Fig 5C). For Redβ at *c*_*β*_ of 100 nM, the measured diameter was ≈4 nm. This diameter is consistent with the size of a Redβ monomer based on its molecular weight (30 kDa) and atomic force microscopy measurements [4]. At concentrations above 100 nM, the average hydrodynamic diameter increased up to ≈10 nm, which is comparable to the previously observed 15-nm diameter of Redβ 11- to 12-mer rings [4,9]. The expected ssDNA diameter for a random ssDNA coil was observed at *c*_*β*_ < 500 nM (Fig 5C, cyan triangles; Section E in S1 Text). Above this concentration, ssDNA association with Redβ was similar to that seen in the gel assay (Fig 5B). To directly verify Redβ–ssDNA binding, we calculated the cross correlation (fluorescence cross correlation spectroscopy [FCCS]) [41], Fig 5C, purple circles) of simultaneously recorded fluorescence signals, which showed significant Redβ–ssDNA binding at 100 nM (≈30%); at ≈300 nM Redβ, half the ssDNA molecules were bound. Together, these data indicate that only Redβ monomers were bound to ssDNA at in vivo concentrations (green line in Fig 5C), further supporting the notion that Redβ monomers drive the onset of filament formation.

### A Quantitative Model Supports Monomer-Driven Annealing

Even though FCS is a single-molecule technique, the hydrodynamic diameter that is measured is an average value. Thus, it is still possible that a small population of Redβ rings, which does not significantly change the average radius, is responsible for annealing. To rule out this possibility, we developed an annealing model based on first principles. We briefly summarize the model in the next paragraph followed by an in-depth account (see also Section F in S1 Text). Readers who are only interested in the essentials of the model can continue with the discussion after reading the following paragraph.

**Fig 6:**
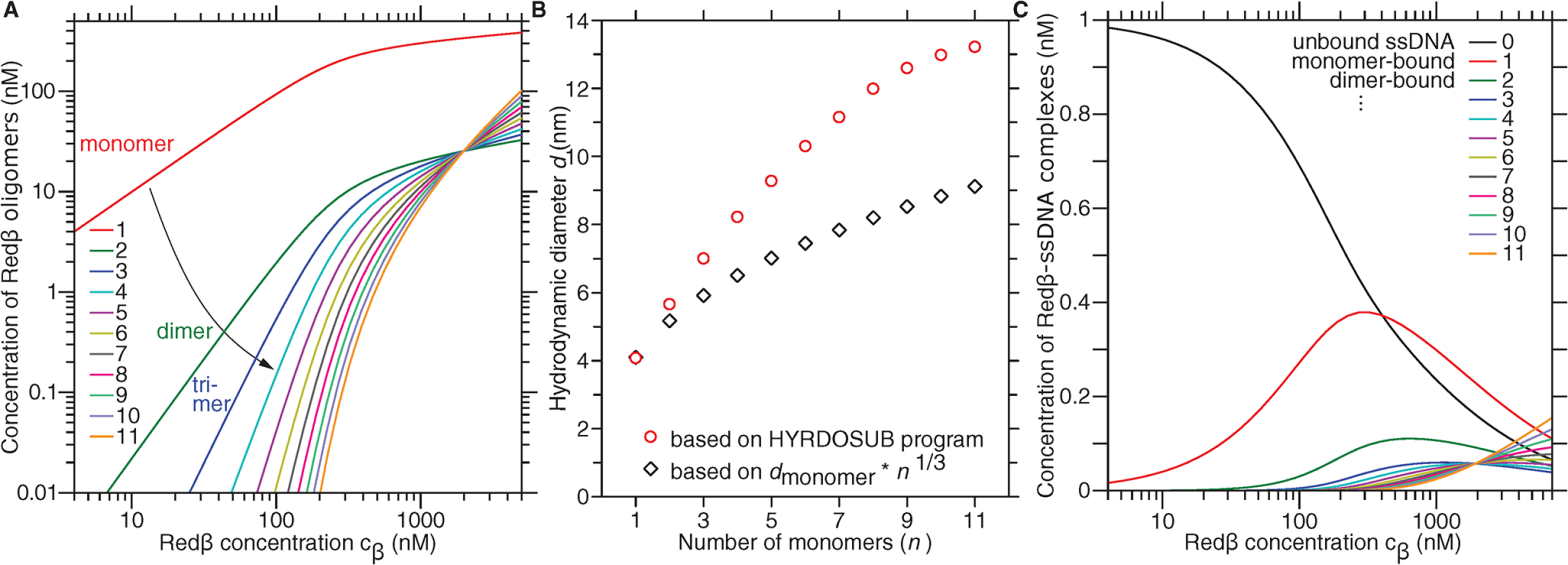
Annealing model. (A) Redβ oligomer concentration calculated according to Eq 4 using the parameter values given in Table 1 plotted versus the total (pipetted) Redβ concentration. All oligomer curves intersect at a Redβ concentration at which the monomer concentration of [*β*_*1*_] = *a* ^*-1*^. (B) Hydrodynamic diameter (red circles) calculated by the software Hydrosub [42] as a function of oligomer size (number of monomers *n*). The diameter of a sphere with the volume of *n* monomers is plotted as a reference (black diamonds). (C) Concentrations of Redβ–ssDNA complexes. The number of Redβ subunits attached directly (i.e., as monomer) or indirectly (i.e., as part of an oligomer) to the ssDNA is indicated by *n* (*n* = 0 corresponds to unbound ssDNA of 30-b length, *n* = 1 is a bound monomer, *n* = 2 can be a bound dimer or two bound monomers, etc.).

To compare the model to the FCS measurements, we had to (i) account for the self-association of Redβ, (ii) compute the hydrodynamic diameter of each oligomeric species of Redβ, (iii) calculate the contribution of each species to the FCS signal, and (iv) quantify the binding equilibrium between Redβ and ssDNA. Only with the complete theory, we were able to fit all the data in a global manner, i.e., with the same basic parameters for all datasets. To this end, we modeled the self-association of Redβ to form different oligomeric species by isodesmic growth (Fig 6A). Based on the computed hydrodynamic diameter of the individual Redβ species (Fig 6B), their relative concentrations according to a nucleated isodesmic growth model and their relative contribution to the FCS signal, we calculated the average diameter as measured by FCS. A best fit to the measured diameter (Fig 5C) favors a model in which a dimer forms the nucleus for growth. In a similar manner, we determined the size of oligonucleotides with different numbers of Redβ molecules bound according to their affinity and concentration (Fig 6C). A global least-square fit to both the FCS data of Redβ and ssDNA, and their FCCS signal showed very good agreement between the data and the model (Fig 5C; compare the data points to the three calculated plotted lines, respectively). The model is consistent with the mechanical (optical trapping), structural (CD), and electrophoretic mobility data, and the best fit of only a few basic parameters provided quantitative binding constants (Table 1). Overall, the model supports the notion that annealing is initiated by Redβ monomers and that filament growth is nucleated by dimerization.

**Table 1:** Constants used for the quantitative model of Redβ behavior.

Constant	Value	Unit	Explanation
*a*_dimer_	(2.22 ± 1.28) ∙ 10^5^	M^−1^	Association constant of *β*_1_ + *β*_1_ ⇆ *β*_2_
*a*	(2.96 ± 0.28) ∙ 10^6^	M^−1^	Association constant of *β*_*n*_ + *β*_1_ ⇆ *β*_*n*+1_, *n* ≥ 2
*K*_*d*_	(5.0 ± 0.2) ∙ 10^−6^	M	Dissociation constant of *β*_1_ + ssDNA ⇆ ssDNA ⋅ *β*_1_
*K*_oligo_	(3.4 ± 0.6) ∙ 10^−1^		Dissociation constant of Redβ protomers *β*_u*nbound*_ ⇆ *β*_b*ound*_ from ssDNA

Parameter ± standard error. Protomer: structural unit of a protein oligomer; *a*_dimer_ = *a*_m = 1_, *a* = *a*_m>1_, where *m* is the degree of oligomerization; see Eq 4. Note that the *K*_*d*_ is for a single binding site. The effective *K*_*d*_ depends on the ssDNA length and, here, is *K*_Epstein_ ≈ 240 nM.

### Redβ Oligomerization Is Described as Isodesmic Growth

To determine the concentration of Redβ monomers, dimers, trimers, and so forth up to ring-like oligomers, we modelled the polymerization. We chose one of the simplest polymerization models that was sufficient to explain our data. In the simplest model, one self-association constant *a* independent of the oligomer size determines the lateral affinity of Redβ. In this isodesmic growth model [43], one subunit—a Redβ monomer *β*_1_—is added at a time to a Redβ oligomer *β*_*n*_ according to the reaction below:

$$\beta_{n}+\beta_{1}⇆\beta_{n+1},$$ (1)

where *n* is used as an index representing the number of Redβ protomers (monomeric subunits of an oligomer). In thermodynamic equilibrium, following the law of mass action, the equilibrium constant for this reaction is given by the following:

$$a=\frac{\left[\beta_{n+1}\right]}{\left[\beta_{n}][\beta_{1}\right]}.$$ (2)

Solving for the concentration of an *n*-mer [*β*_*n*_] results in

$$[\beta_{n}]=a^{-1}\;{\left(a[\beta_{1}]\right)}^{n}.$$ (3)

If *a* is not constant but depends on the degree of oligomerization *m*, [*β*_*n*_] is determined by the product of the association constants *a*_*m*_:

$$[\beta_{n}]={\left[\beta_{1}\right]}^{n}\prod_{m=1}^{n-1}a_{m}.$$ (4)

In either case, the total concentration of Redβ molecules [*β*], i.e., the pipetted Redβ concentration, is given by the following:

$$[\beta ]=\sum_{n=1}^{11}n[\beta_{n}],$$ (5)

where we limited the maximum number of Redβ molecules in an oligomer to *n* = 11 based on the ring-like Redβ structure determined by atomic force microscopy [4]. Even though this structure is an open helical ring, the pitch of the helix does not allow larger oligomers. Also, we did not consider the formation of larger structures like stacks of rings. For a given pipetted concentration [*β*] and association constants *a*_*m*_, we numerically determined the monomer concentration [*β*_1_] from the total-concentration equation Eq 5 and, subsequently, the oligomer concentration using Eq 4 (Fig 6A). The best fit of the model to the data suggested a dimer-nucleated process, i.e., the fit to the data was significantly improved when we chose two constants instead of a single association constant (Fig 5C, Table 1). Since Redβ is known to nucleate the polymerization of a nucleoprotein filament [9,44], nucleation is a reasonable assumption.

### The Model Is Consistent with the FCS-Measured Hydrodynamic Diameters and the Cross Correlation

Based on the structure and the relative concentration of Redβ oligomers, we calculated the average hydrodynamic diameter and compared it to the FCS measurements. The structural parameters of Redβ oligomers have been measured by atomic force microscopy: monomers self-associate to eventually form a “split washer” [4]. The calculated hydrodynamic diameter *d* (circles in Fig 6B) increased from 4.1 nm for the monomer to about 13 nm for an 11-mer. This diameter is reasonable since it is larger compared to a sphere with the volume of 11 monomers (≈9 nm, diamonds in Fig 6B) and smaller compared to the outer diameter of the ring-like complex (≈18 nm). To account for the mixture of Redβ species, we calculated an average hydrodynamic diameter. We weighted the diameter of an oligomer species by its relative concentration (Eq 4, Fig 6B) and a size-dependent brightness factor (Section F in S1 Text). The best fit to the data (red solid line Fig 5C) shows very good agreement and supports the model.

To determine how the number of bound Redβ molecules per ssDNA and the hydrodynamic diameter of Redβ-bound ssDNA scaled with the pipetted Redβ concentration, we model the interaction as shown below:

$$\beta_{n}+\text{ssDNA}⇄\text{ssDNA}\cdot \beta_{n}.$$ (6)

The equilibrium or dissociation constant for a Redβ-*n*-mer bound to ssDNA is denoted by *K*_*n*_, given by the following:

$$K_{n}=\frac{\left[\beta_{n}][\text{ssDNA}\right]}{\left[\text{ssDNA}\cdot \beta_{n}\right]}\;,$$ (7)

where the square brackets denote the concentration of the respective species. If the binding interface between every Redβ molecule and ssDNA is identical, the binding energy must also be equal. Thus, to a first approximation, the dissociation constant is $K_{n}=K_{d}^{n}$, where *K*_*d*_ is the dissociation constant for a Redβ monomer and ssDNA. However, since entropic effects and mutual interactions between Redβ molecules may change the free energy, we modelled the constant as $K_{n}=K_{d}K_{\text{oligo}}^{n-1}$, where we distinguished a monomeric, *K*_*d*_, and protomeric, i.e., the subunit of an oligomer, *K*_oligo_, dissociation constant. To account for multiple monomers as well as oligomers binding simultaneously to ssDNA, we determined all possibilities according to the theory of Epstein [45] (Section F and Table C in S1 Text). Thus, using (i) the concentrations of the respective species obtained from the isodesmic growth model, (ii) the number of bases bound per Redβ (*n* = 10 based on our data of Fig 2D), and (iii) the equilibrium constants *K*_*d*_ and *K*_oligo_, we calculated how many Redβ molecules were bound to what fraction of ssDNA molecules (Fig 6C). The parameters for the calculations are based on a best fit to the FCCS data (purple line in Fig 5C). With increasing Redβ concentration, the concentration of unbound ssDNA decreased. Interestingly, monomer-bound ssDNA reached a maximum at *c*_*β*_ ≈ 300 nM, coinciding with the half occupancy measured via FCCS (Fig 5C). The concentration increase of the ring-bound fraction (11-mers) coincides with the decrease in filament yield, which we observed in the EMSAs. At in vivo Redβ concentrations, the ring-bound ssDNA concentration is calculated to be below 0.1 nM. Thus, in *E*. *coli* not even a single ring should exist either in solution or bound to ssDNA. Because of these low concentrations and the law of mass action (see also third paragraph of the Discussion below), it is unlikely that rings are responsible for SSA.

Knowing how many Redβ molecules are bound per DNA as a function of Redβ concentration, we could calculate the average hydrodynamic diameter of Redβ–ssDNA complexes as measured by FCS. First, we calculated the expected hydrodynamic radius of freely diffusing ssDNA (Section E in S1 Text). Second, to determine the increase in the apparent ssDNA diameter as a function of Redβ concentration in Fig 5B, we assumed that the hydrodynamic diameter of a Redβ-bound ssDNA, ssDNA∙*β*_*m*_, corresponded to the diameter of a Redβ oligomer *β*_*m*_ as numerically determined in Fig 6B. We then performed a weighted average using the relative concentration of the individual species and a size-dependent brightness factor resulting in the curve (cyan line) plotted in Fig 5C. Considering that Redβ-bound ssDNA exhibits considerable flexibility and heterogeneity [4], the agreement between calculation and measurements is remarkable.

## Discussion

**Fig 7:**
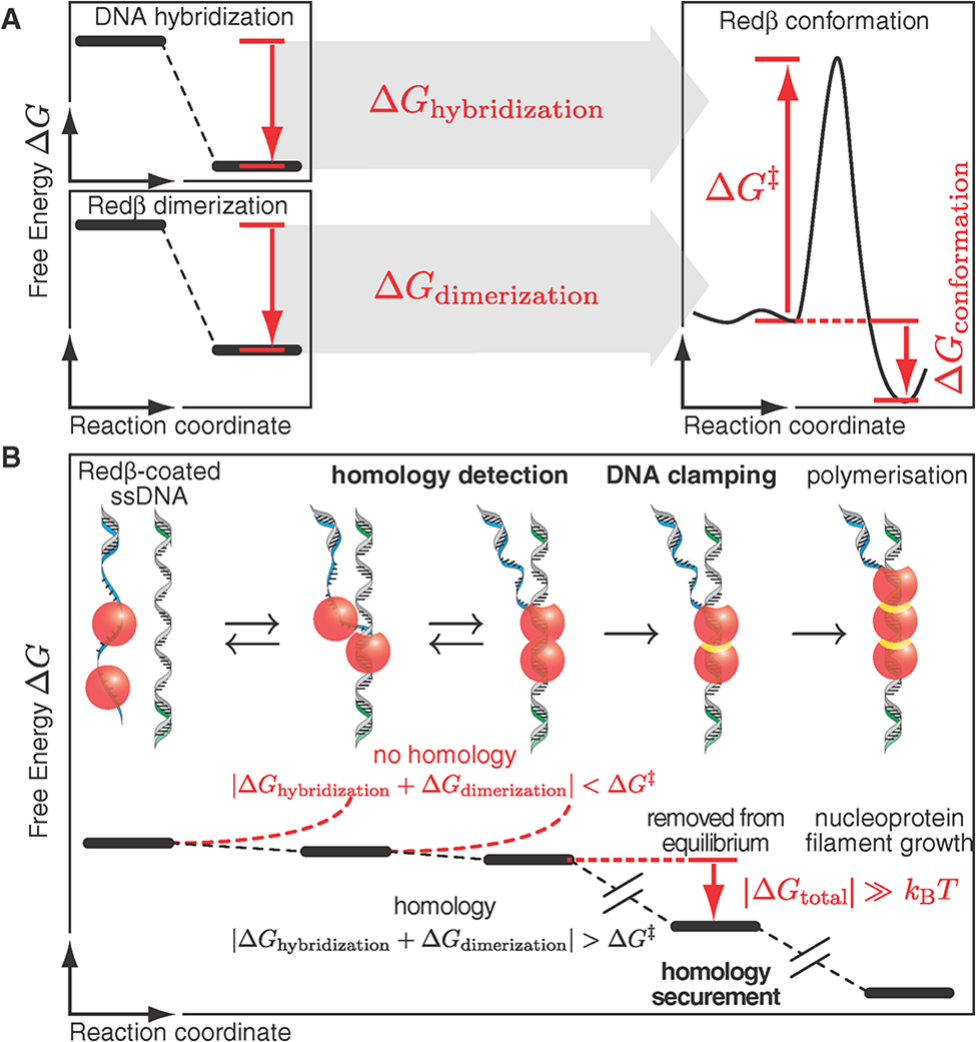
Faithful homology detection and securement. (A) Interaction of ssDNA-bound Redβ with a complementary strand is hindered by an energetic barrier ∆*G* ^‡^. Hybridization of matching DNA strands (∆*G*_hybridization_) together with dimerization (lateral association) of ssDNA-bound Redβ (∆*G*_dimerization_) overcomes this barrier to form a stable nucleoprotein complex (∆*G*_conformation_). The differences in free energies ∆*G*_dimerization_, ∆*G*_hybridization_, and ∆*G*_conformation_ result from the difference between the initial and final state along the reaction coordinate. For clarity, further factors such as thermal fluctuations were omitted. (B) To overcome the energetic barrier ∆*G* ^‡^ and trigger the conformational change for DNA clamping, sufficient free energy (| Δ*G*_h*ybridization*_ + Δ*G*_d*imerization*_ + Δ*G* ^‡^ |≈*k*_B_ *T*) can only be provided by sufficient DNA sequence homology, where *k*_B_ *T* is the thermal energy (*k*_B_: Boltzmann constant, *T*: absolute temperature). The total free energy gain ∆*G*_total_ = ∆*G*_hybridization_ + ∆*G*_dimerization_ + ∆*G*_conformation_ is much larger than the thermal energy, making clamping sufficiently stable. The clamped state of DNA-bound Redβ with a lateral contact is marked with a yellow line.

How does Redβ detect homology with high fidelity (Fig 7)? The mechanism is initiated with the association of Redβ monomers and oligomers with ssDNA, with a moderate preference for 3′-ended ssDNA as released by its partner exonuclease Redα [4]. If an associated monomer finds a complementary sequence, it weakly holds 10 ± 2 bases together (Figs 1 and 2). However, this homology length is not unique in the *E*. *coli* genome with a total length of *L* = 4.6 Mb, giving a 1 − (1 + γ)*e* ^−γ^ = 93% chance (where the factor γ is given by γ = *L*/4^*n*^), according to Poisson statistics, of at least two *n* = 10 nt regions of equal sequence. Unique recognition is only ensured if at least two neighboring Redβ molecules bind complementary DNA. Thus, annealing proceeds when a second Redβ molecule enters the complex and also binds complementary DNA. Conformational proofreading has been suggested for homology detection [46]. In this model, the binding probability of complementary ssDNA is reduced in the presence of a repair protein. This reduction, associated with a reduced annealing rate, is caused by a conformational change, e.g., a change in the distance between bases, upon protein binding. Such a scenario is advantageous because only near-complete complementarity can provide enough energy for annealing. In case of a mismatch, thermal energy should be sufficient to dissociate bound proteins. Our data provide some evidence that Redβ follows a conformational proofreading mechanism. During hairpin annealing, we observed a hysteresis and a drop in force prior to unzipping peaks. The annealing impairment is consistent with a reduced binding probability of complementary ssDNA. Upon Redβ binding, a change to the conformation of the ssDNA will reduce the DNA annealing rate and cause a drop in force. Such a conformational change is also supported by our CD data: the filament contained dsDNA close to B-form DNA. This structure may indicate that the distance between bases of Redβ-bound ssDNA is smaller compared to unbound ssDNA, explaining the increased annealing hysteresis by conformational proofreading [32,47]. If Redβ would not change the conformation of the bound ssDNA, the energy of a single mismatch could not compete with the large hybridization energy of the remaining Redβ-bound bases with a complementary strand. If a neighboring Redβ molecule binds and homology is given, the energetic gain of base-pairing, the lateral contact (Fig 3) and association by dimerization, and the conformational change (Fig 4) cause an irreversible “decision” to proceed with nucleoprotein filament growth and polymerization. In terms of energy, this decision must correspond to a large, virtually irreversible, reduction in free energy as evidenced by the strong DNA clamping (Fig 2) and structural change (Fig 4). Our model assumes that a certain amount of energy is necessary to convert Redβ from the nonclamped state into the clamped state, making correct annealing and DNA clamping a coupled process. The energetic barrier of the transition provides insurance against recombination of nonhomologous sequences, because without annealing, insufficient energy is available to cross the activation barrier before the Redβ-bound strands dissociate again because of thermal energy.

Our data show that lateral interactions between the monomeric subunits of a nucleoprotein filament resist external forces (Fig 3). We attribute a crucial role to this lateral interaction because its free energy (∆*G*_dimerization_) together with the hybridization energy (∆*G*_hybridization_) is necessary to overcome the energetic decision barrier of recombination ∆*G* ^‡^, ensuring a sufficient kinetic delay in case of sequence mismatches. Only in the absence of a mismatch can Redβ reside long enough at a certain site and in the right conformation to allow another Redβ molecule to dimerize. Thus, it is advantageous if the on-rate for dimerization is lower than the on-rate for nucleoprotein filament growth to assure that Redβ can find a matching site. Only in this case will the dimerization be sensitive enough to respond to a mismatch. A strong affinity in the first dimerization step would lead to filament growth at the site of first encounter with near complementarity, i.e., to a larger, unfavorable mismatch tolerance. A long period for site selection followed by rapid stabilization due to the oligomerization would ensure faithful recombination. While on the filament itself we do not know these rates, in solution our model provided a lower association constant of the initial dimerization compared to larger complexes. This may be an indication that a similar on-rate ratio exists on the filament. Apart from this dynamic aspect, the lateral interaction ensures that monomeric subunits are aligned in a juxtaposed fashion. In this manner, a regular nucleoprotein filament will grow as observed experimentally. The lateral interaction also ensures that a continuous sequence is compared, i.e., two laterally associated Redβ molecules scan 20 continuous bases. Thus, in the absence of DNA, the lateral assembly of protein monomers into quaternary structures—such as rings—appears to be a side effect related to the energetic contribution of dimerization rather than the source of an active oligomeric species. This conclusion provides a rationale for the notion that high protein concentrations, which favor rings and other quaternary structures, are detrimental to strand annealing reactions [12–15].

Our model favors the view that annealing is promoted by Redβ monomers. Based on the best-fit parameters, the monomeric Redβ concentration saturates with increasing Redβ concentration *c*_*β*_ (Fig 6A). In a single *E*. *coli* cell (*c*_*β*_ ≈ 90−140 nM), not even a single ring (*n* = 11) should be bound to ssDNA or even exist in solution. Reported images for ring-like Redβ structures required more than 0.8 μM of protein and often involved longer periods of incubation with glutaraldehyde for fixation [4,9,16], which shifts the chemical equilibrium towards ring-like structures. In our model for *c*_*β*_ < 1,000 nM, only smaller oligomers (*n* < 7) are present in concentrations larger than 10 nM. At *c*_*β*_ = 1,000 nM, the ring concentration (*n* = 11) is about 50-fold smaller compared to the Redβ monomer concentration and at the half-occupancy concentration of ssDNA of *c*_*β*_ = 300 nM more than three orders of magnitude (≈2,000-fold) smaller. Between these two Redβ concentrations, there is about a 40-fold (2,000/50) increase in the ring concentration. If the ring constitutes the nucleus of the annealing reaction, one would also expect about a 40-fold increase in reaction products based on the law of mass action. However, there is only about a 2-fold increase in filament yield (Fig 5C). Thus, for *c*_*β*_ < 1,000 nM, the ring-like structures are unlikely to be the decisive component for the annealing reaction. Models that predict higher ring concentrations at low Redβ concentrations are inconsistent with our FCS data. Significant ring concentrations occurring at *c*_*β*_ > 1,000 nM correlate with impaired filament formation (Fig 5C). This decrease is consistent with previous reports about Redβ and related proteins [5,9,11–13,48,49] and suggests that ssDNA bound to rings is nonreactive, i.e., it cannot anneal to a complementary strand. It implies that at high Redβ concentrations, which favor ring-like complexes, Redβ does not promote annealing but instead impairs it.

Moreover, the fluorescence cross correlation data support monomer-driven annealing. Fitting a Hill equation to the cross correlation data resulted in a Hill coefficient close to 1 (Section F in S1 Text). A coefficient close to unity implies only little or no cooperativity—for ring-binding, we would expect a Hill coefficient of up to 11. A coefficient not significantly different from 1 also suggests Michaelis-Menten–like binding of Redβ monomers to ssDNA. Interestingly, when we compared the percentage of Redβ-bound ssDNAs to the FCCS measurements (Fig 5C), i.e., for *c*_*β*_ ≲ 500 nM, the cross correlation was mainly accounted for by monomers binding to ssDNA (*n* = 1 in Fig 6C). Indeed, a fit of a Michaelis-Menten–like equation to the FCCS data resulted in a dissociation constant consistent with (i) our model-based, best-fit value (Table 1) and (ii) a rough estimate from the literature [39]. Note that even though Redβ’s *K*_*d*_ with ssDNA for a single binding site is about 5,000 nM (Table 1), the effective *K*_*D*_ according to the Epstein model is much lower because of multiple binding sites. For a 30-b-long ssDNA, the constant is *K*_Epstein_ ≈ 240 nM—of similar magnitude as the in vivo concentration. Thus, for DNA repair, for which a single break would correspond to a concentration in the 1 nM range, the Redβ–ssDNA affinity is high enough for an effective repair mechanism.

How can the nucleoprotein filament be eventually disassembled in vivo? The high stability of the filaments against force also implies a very small filament-disassembly rate in the absence of force, as evidenced by the sharp filament bands in nondenaturing gels (Fig 2F and inset of Fig 5C) and the ability to image such filaments with atomic force microscopy in the absence of fixation [4]. The unzipping of pure dsDNA requires about 15 pN (Fig 1), in agreement with earlier experiments [50,51]. The exceptionally large unzipping forces of the nucleoprotein filament exceeding 200 pN (Fig 2) under low loading-rate conditions (≲ 40 pN/s) are surprising because such large forces have only been reported for protein unfolding or amyloid fibril disruption: for the unfolding of immunoglobulin domains, forces of 150–300 pN have been measured [52], about 140 pN for tenascin [53] and up to 250 pN for amyloid fibrils [54]. As a comparison, the strongest reported biological motor can generate forces of about 60 pN [55], which equals the force necessary for force-induced DNA melting [24]. As shown for RecA and Rad51 nucleoprotein filament disassembly by UvrD [56,57] and Srs2 helicase [58,59], respectively, or adenosine triphosphate (ATP) hydrolysis [60,61], we suggest that the Redβ filament may be unraveled by a dedicated helicase or another molecular machine that circumvents the need to exert high force. For efficient recombineering, filament disassembly after annealing is required and must be accomplished by the host machinery by a yet unknown mechanism.

Do strand annealing and strand invasion share mechanistic properties? A major difference between strand invasion and strand annealing is the target substrate. To allow homology detection by Watson-Crick base pairing [62] in strand invasion, the DNA strands of the double-stranded target have to be melted by overstretching [63,64]—a process not necessary in strand annealing. In strand invasion, the energetic coupling of DNA melting with the homology search improves the detection performance by imposing an energetic barrier, which can be best overcome by the hybridization of mismatch-free DNA strands, leading to efficient exclusion of mismatches by conformational proofreading [46]. For strand annealing, a conformational change of the strand-annealing protein coupled to hybridization and dimerization allows the fine-tuning of the homology detection, again in agreement with conformational proofreading. Apart from the different substrates, there are remarkable biochemical commonalities, including the aforementioned assembly into quaternary structures in the absence of DNA [65,66] as well as reaction impairment at high protein concentrations [67,68]. Notably, for the process of homology recognition, neither strand invasion nor strand annealing require the hydrolysis of a high-energy substrate, such as ATP [69,70]. For strand invasion, ATP hydrolysis drives the disassembly of nucleoprotein complexes or elongates the filament past heterologous sequences [71]. Consequently, theoretical models describe homology detection as an equilibrium process taking place without additional energetic input [46,72]. Based on the similarity of biochemical data between strand annealing and strand invasion and considering that both utilize protein–DNA interactions for the detection of matching DNA sequences, we suggest that strand invasion and strand annealing share the same underlying physical mechanism of detecting and securing homology. In addition to the conformational proofreading mechanism for homology detection, we propose a coupled process in which an activation barrier is crossed to reach a final, conformationally stable, i.e., irreversible or sufficiently stable, state upon correctly having identified homology. The latter step is an important and essential addition to current equilibrium detection models. In conceptual agreement with this suggestion, some strand-annealing proteins have been reported to show strand-invasion activity [49,73] or aid the displacement of DNA strands [44,74]. The homology search in thermal equilibrium alone does not account well for such a finding, because it does not include a dedicated mechanism for securing homology, i.e., the removal from equilibrium (Fig 7). DNA clamping provides a rationale for this observation, because—as the product of a directed reaction—the nucleoprotein filament is energetically more stable than the competing dsDNA. Notably, for both strand invasion and annealing, the reported length of the minimum effective processing segment falls in the range of 20–30 bp [4,75–77], with a longer segment in case of RAD51 being attributed to auxiliary proteins and not the homology detection machinery [78].

Both our in vitro biochemical and single-molecule data support a Redβ monomer-driven annealing mechanism via DNA clamping upon Redβ dimerization. In vivo, this annealing mechanism should be optimal at a Redβ concentration of *c*_*β*_ ≈ 300 nM, as suggested by the FCCS-measured half occupancy of ssDNA with Redβ (Fig 5B). Indeed, we measured an in vivo Redβ concentration of *c*_*β*_ ≈ 110 nM (Fig 5C) indicating that also under in vivo conditions, single-strand annealing (SSA) likely is promoted by Redβ monomers. At this concentration, Redβ–ssDNA binding reaches half occupancy (purple line in Fig 5B), and the amount of Redβ bound to ssDNA can be regulated efficiently by changes of the Redβ concentration. Furthermore, our model concords with the applied properties during Redβ-mediated recombineering [35,79–81], which has established that the lower limit of homology for productive recombination is about 25 bp, corresponding to dimer annealing [4]. Apart from occluding false positives during annealing, do the unusually large DNA clamping forces have another in vivo implication? In Redβ-related eukaryotic proteins such as RAD52 [4,5,33,82], a large dissociation force of a clamped state could serve a biological function by withstanding chromosome segregation forces in the presence of dsDNA breaks [2,83,84]. Because other SSAPs share similar biochemical properties with Redβ [12,13,48], we suggest that the general process underlying the initiation of homologous recombination by DNA annealing is due to the action of protein monomers, whose polymerization on DNA by clamping the DNA strands together ensures faithful recombination.

## Materials and Methods

### Buffer Conditions

If not noted otherwise, the standard buffer used for our experiments had the following composition: 20 mM Tris-HCl, pH = 7.5, 10 mM MgCl_2_, and 10 mM NaCl, 0.1% (vol/vol) Tween 20. All DNA concentrations are stated for molecules and not nucleotides.

### Optical Tweezers Experiments

The optical tweezers and calibration procedures have been described in detail previously [18–22]. Optical tweezers experiments were carried out in a microfluidic sample chamber assembled from a 22 × 22 mm microscope cover slip (Corning, #1.5), forming the bottom surface, and an 18 × 18 mm cover slip (Menzel, #1), forming the top. The bottom cover slip was spin coated for 25 s at 10,000 rpm and 2,500 rpm/s acceleration, applying a solution of 0.1% (w/w) polytetrafluoroethylene (CAS 9002-84-0) in Fluorinert FC-75 (Acros Organics) under a nitrogen atmosphere. A cavity of 100 μm height was formed by putting two stripes of double-sided sticky tape (Lehmann DuploCOLL 3720) at a distance of 3 mm in parallel between the cover slips. Thin pipette tips (Eppendorf GELoader) were pressed at their ends with a 125°C hot brass stamp and attached to opposite outlets of the chamber. Thereafter, the chamber was sealed with epoxy glue (5 Minuten-Epoxy, R&G Faserverbundwerkstoffe Waldenbuch). The flow cell was incubated for 30 min with 20 ng/μl antidigoxigenin antibodies for passive adsorption to polytetrafluoroethylene. The antibody solution was removed with blocking solution containing 0.1 mg/ml bovine serum albumin (New England Biolabs) and 0.01% (m/vol) F127. To maximize the trap stiffness with the available laser power, we optimized the laser expansion to slight underfilling [22] and chose a microsphere size close to the first Mie resonance [22,85,86]. In this manner, we could achieve trapping forces of up to 600 pN using polystyrene microspheres [86]. We used carboxylated microspheres of 920-nm diameter (Bangslabs) for carbodiimide coupling of NeutrAvidin. By laterally moving the sample stage, a 3,562-bp dsDNA was unzipped with one strand ligated to a 944-bp digoxigenin-terminated dsDNA anchor attached to the antidigoxigenin incubated flow cell surface and the other to a 441-bp biotin-terminated dsDNA anchor attached to the neutravidin-coated microsphere. We used *c*_*β*_ = 2 μM. Loading rates were about 1,000 pN/s, trap stiffnesses were 0.9, 1.0, and 0.4 pN/nm in the *x*-, *y*-, and *z*-axis, respectively. The same construct with either a 25-bp or 123-bp dsDNA in the middle was used for unzipping of the nucleoprotein filament. The latter two were reinforced with additional 267-bp-long dsDNA anchors produced by PCR with added dU-biotin and dU-digoxigenin. The 25-bp or 123-bp constructs were assembled by incubating its 3′ hydroxyl overhang strand with 750-nM Redβ, followed by addition of the strand containing the complementary overhang. All constructs have ssDNA spacers on each side of the unzippable portion. For the nick-sealing experiments, PCR-produced biotin- and digoxigenin-containing anchors had been used as described above. For the 25-bp and 123-bp constructs, the loading rate was 100 pN/s. For the 123-bp construct, the force was kept constant by a software-based, three-dimensional force feedback operated at 1 kHz using the sample piezo-translation stage. The setup has sub-nm precision with mK temperature-controlled objectives [20]. For all trapping experiments, the objective temperature was 29.2°C. To dissociate the filament, we ramped up the force to a certain threshold value. Once this value was reached, the feedback automatically engaged and kept the force constant. If no dissociation event was observed within a few seconds, the force was ramped up to a new threshold value until eventually dissociation steps were observed. We assigned the steps with an automatic step finder [21]. For the step-size conversion based on the extensible freely jointed chain model [87], we used a contour length of *L*_0_ = 0.44 nm/base, a persistence length of 0.73 nm, and an elastic modulus of 840 pN (see Section E in S1 Text). To quantify the amount of hysteresis as a function of time in the presence of Redβ, we numerically calculated the area *A* between the stretch and release curve (Fig 3B) and the area *B* between the release and theoretical ssDNA force-extension curve with the above-mentioned model. The ratio *r* = *B*/(*A* + *B*) is less than 1 with hysteresis (Fig 3C, equivalent to the control of Fig BB in S1 Text) and approaches 1 without hysteresis (Fig 3C, equivalent to the control of Fig BC in S1 Text). Since our controls without Redβ (Fig BB and BC in S1 Text) verified that hysteresis is due to DNA strands with unconstrained ends peeling off, we attribute the reduction of hysteresis to the action of Redβ clamping the strands together and preventing them from coming apart.

### CD

CD was measured with a Jasco J-815 system. Aqua bidest. (*ρ* = 18.2 MΩcm) was used as the baseline. 16 spectra were recorded under nitrogen atmosphere at 1-mm path length, 100-nm/min scanning speed, 20°C temperature, and 5-nm bandwidth. The spectra were averaged. Absorbance spectra and the high tension voltage applied to the dynodes were concurrently recorded during all measurements. For the generation of the nucleoprotein filament, 250 nM of a 44-b-long ssDNA_CD_(1) (Table B in S1 Text) was incubated with 1 μM Redβ for 20 min and mixed with 250 nM of a complementary ssDNA_CD_(2) of equal length. The incubation time after addition of the complementary strand was 1 h. The solution with an initial volume of 5 mL was concentrated about 10-fold by water evaporation at low pressure and at 25°C. For the control “Redβ,dsDNA,” the same ingredients were used, but the complementary ssDNAs were mixed and annealed (temperature ramp starting from 60°C, cooling 1 K/min to 4°C) before addition of Redβ. The final volumes were adjusted with aqua bidest. to match the 260-nm absorbance of 1 μM dsDNA (prepared as described above). The control “Redβ,ssDNA” was prepared as the filament, but without ssDNA_CD_(2).

### Western Blot Analysis of Redβ In Vivo Concentration

Using the reference bands, the amount of protein was calculated for the sample bands. To quantify the western blot band intensities (Fig 5C), we placed a rectangular selection on the reference bands containing a known amount of Redβ using the software ImageJ [88]. Since black corresponds to a low value, the images were inverted prior to analysis to correlate increasing band strengths with increasing values. Using the integrated density of the reference bands, we determined the intensity change per nanogram. Accounting for the background, the integrated density of the 40-min lane corresponded to 28, 36, 46, and 36 ng for the four independent measurements, respectively. Based on our reference count of 8 ∙ 10^8^ cells/ml corresponding to an OD_600_ = 1.0, there were 2.9 ∙ 10^9^ cells used for the 40-min induction lanes. Assuming a volume of a single GB2005 cell of 3.8 μm^3^ [38], the total cell volume was 2.9 ∙10^9^ × 3.8 μm^3^ ≈ 11 μl. Based on the concentration of *E*. *coli* determined via the OD measurement, the used volume, and the molecular weight of Redβ of 29,689 g mol^-1^, the above values correspond to ≈86, 110, 141, and 110 nM, respectively, with a mean ± SE of 110 ± 10 nM or ≈250 molecules of Redβ per *E*. *coli* cell.

### Native Polyacrylamide Gel Electrophoresis

The native polyacrylamide gels used for Redβ and DNA band-shift assays were cast according to standard procedures. For a final volume of the separating gel of 50 ml (20 ml of the stacking portion) the following components were mixed: 40% Acrylamide (19:1 Bis.) 10 ml (2.5 ml), 10x TBE 12.5 ml (2.5 ml), H_2_O 27 ml (14.8 ml), TEMED 30 μl (20 μl), 10% APS 500 μl (200 μl). The samples contained 25 fmol of 30-b-long Atto 565-ssDNA_B_ (Table B in S1 Text) and Redβ with a constant 5 nM chemically labelled with Alexa 488 using the Alexa Fluor 488 Microscale Protein Labeling Kit (Invitrogen). Prior to application on the gel, the ssDNA was incubated with Redβ for 20 min. For filament formation, 1 nM of a 30-b-long 5′ Atto 565-labelled and 0.67 nM of a 50-b-long ssDNA were incubated for 20 min with Redβ and united, followed by 15 min of incubation, producing a 5′ overhang. A saturated sucrose solution was mixed with the sample in a ratio of 1:6 (vol/vol) to increase its density for loading. The samples were applied to a 5% stacking and 8% separating gel and run for 120 min at 100 V at 4°C in 1 × TBE buffer. The gel pictures were recorded with a Typhoon 9410 Imager. To visualize the bands of Fig 2 using ethidium bromide staining, we used a ssDNA concentration of 50 μM. Accordingly, we used a high concentration of unlabeled Redβ of 100 μM.

### FCS

FCS was conducted using a ConfoCor3/LSM510 (Zeiss) with a Zeiss C-Apochromat 40× (NA = 1.2) water immersion objective. 488-nm and 633-nm laser lines were used to excite Alexa 488-tagged Redβ (labeling ratio 3 Alexa 488 per 1 Redβ) and Atto 655-tagged ssDNA of 30-b length. Fluorescence was restricted to the plane of interest using a pinhole with 70-μm diameter. The signals were correlated with the ConfoCor3/LSM510 product software and fitted with a weighted Levenberg-Marquardt routine. Calibration of the excited volume was done using the unbound dyes Alexa 488 (*D* = 433 μm^2^/s) and Atto 655-COOH (*D* = 427 μm^2^/s), respectively [89,90]. Each data point is based on 30 runs of 20 s duration each.

## Supporting Information

S1 Data All the numerical data. (XLSX)

S1 Text Supporting information containing six text sections, four figures, three tables, and ten references [91–100]. (PDF)

## Abbreviations

ATP: adenosine triphosphate
CD: circular dichroism
DSBR: double-strand break repair
dsDNA: double-stranded DNA
EMSA: electrophoretic mobility shift assay
FCCS: fluorescence cross correlation spectroscopy
FCS: fluorescence correlation spectroscopy
SSA: single-strand annealing
SSAP: single-strand annealing protein
ssDNA: single-stranded DNA
