# Supplementary material for: A Single-Strand Annealing Protein Clamps DNA to Detect and Secure Homology
Source: PLoS Biol. 2015 Aug 13;13(8):e1002213. doi: 10.1371/journal.pbio.1002213 (PMC4535883; doi:10.1371/journal.pbio.1002213)
Supplement: S1 Text — (PDF) [file pbio.1002213.s002.pdf]

SUPPORTING INFORMATION

A Single-Strand Annealing Protein Clamps DNA to Detect and Secure Homology

Marcel Ander[1], Sivaraman Subramaniam[2], Karim Fahmy[3], A. Francis Stewart[2,*], Erik Schäffer[1,4,*]

1 Nanomechanics Group, Biotechnology Center, TU Dresden, Dresden, Germany, **2** Department of Genomics, Biotechnology Center, TU Dresden, Dresden, Germany, **3** Division of Biophysics, Institute of Resource Ecology, Helmholtz-Zentrum Dresden-Rossendorf, Dresden, Germany, **4** Cellular Nanoscience, Center for Plant Molecular Biology (ZMBP), Universität Tübingen, Tübingen, Germany

* francis.stewart@biotec.tu-dresden.de (AFS) and * erik.schaeffer@uni-tuebingen.de (ES)

Contents

A DNA Constructs for Optical Tweezers Assays 2
B Controls Confirm that Redβ Reduced Annealing Hysteresis 2
C Circular Dichroism (CD) Measurements Support a Structural Change During Filament Formation. 4
D Redβ Homo-Oligomerizes 5
E FCS Hydrodynamic Diameter of ssDNA and Force-Extension Measurements Are Consistent with Theory 6
F Annealing Model 7

List of Figures

A Structures of the DNA constructs used for the unzipping experiments. . . . 2
B Controls of dsDNA force-extension behavior without Redβ. 4
C Nucleoprotein filament CD spectrum cannot be described as a sum of the individual components. 5
D Redβ oligomers separated by non-denaturing, polyacrylamide gel electrophoresis. 6

List of Tables

A DNA oligonucleotides used for the optical tweezers experiments.. 3
B Single-stranded DNAs used for the CD, EMSA, and FCS experiments. 5
C Combinatorial binding model. 8

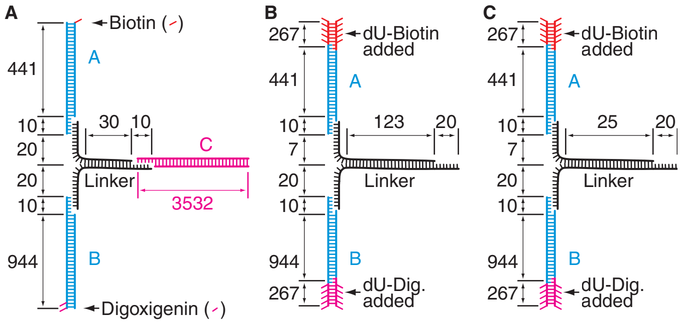

Fig A. Structures of the DNA constructs used for the unzipping experiments. A Schematic showing the three different segments A, B, C, and the "Linker"-segment, which are ligated for the hairpin unzipping experiments. The top and bottom termini are attached to the microsphere and the surface of the sample chamber, respectively. **B,C** For the high-force constructs, the linker consisted of a 123 bp and 25 bp long complementary region without an additional segment C.

A DNA Constructs for Optical Tweezers Assays

Three DNA constructs were used for the DNA unzipping assays: One for hairpin unzipping and two for high-force clamping experiments (Fig. A). Sequences are given in Table A. For PCR, lambda DNA was used as template. The hairpin-unzipping constructs consisted of a 3532 bp-long complementary region, which was unzipped during the experiments (segment C in Fig. AA). This region was linked to two spacer strands (A and B in Fig. AA) that are anchored to the surface of the sample chamber and the trapped microsphere, respectively. Segments A and B were produced using biotin- and digoxigenin-modified PCR primers. The two single strands that make up the segment called "Linker" in Fig. AA were mixed in a 1:1 ratio and annealed by a stepwise decrease of temperature by 1 °C/min, from 65 °C to 4 °C. The annealed linker was mixed with the segments A and B in a ratio of 2.5:2.5:1 (A:B:Linker), ligated, and gel-purified. Subsequently, the band corresponding to the A–B–Linker molecule was cut out from the gel, ligated with C, gel-purified, and cut out again. For high-force measurements (Fig. 2), the complementary region of the linker was 123 bp or 25 bp long. For these constructs, segments A and B were produced by ligation of a 441 and 944 long dsDNA, respectively. The terminal 267 bp of these dsDNAs contained dU-biotin and dU-digoxigenin (Jena Bioscience, Germany) for segment A and B, respectively, incorporated by PCR following the protocol of the supplier. Termini for ligation were produced with primers, which introduced either Nt.BstNBI or Nb.BsrDI (New England Biolabs) cleavage sites at a distance of 10 b away from the end of the molecule and digested at 60 °C. The construct for the nick-sealing experiments was produced in an analogous manner with the primers given in Table A. For the dsDNA with two detachable termini (Fig. BB), the PCR product was digested with EcoRI (New England Biolabs) overnight and purified. The overhang was filled with dA-biotin and dU-biotin using Klenow fragment (3'→5' exo^{-}) (New England Biolabs). For hairpin unzipping experiments, the bottom surface of the sample chamber was spin coated with PFC 504A-coE5 (Cytonix, Beltsville, USA) introducing epoxy groups. The DNA was reacted with the epoxy groups at pH 10.5 for 15 minutes at room temperature and the sample chamber then washed with standard buffer (see Methods).

B Controls Confirm that Redβ Reduced Annealing Hysteresis

To confirm the action of Redβ during force-extension measurements of photo-damaged dsDNA (Fig. 3), we performed several controls with various DNA constructs (Table A) both in the absence and presence of Redβ (Fig. B). First, without Redβ, we used the same construct as in Fig. 3 to confirm the high-force overstretching plateau above 100 pN and, upon photo damage, the subsequent force-drop and hysteresis (Fig. BA). The high-force plateau due to torsionally constrained DNA is in agreement with previous measurements [28, 30]. The force drop to

Table A. DNA oligonucleotides used for the optical tweezers experiments.

Part	Sequence (5' → 3')
DNA unzipping	
A forward	ATACTAAACATAATGACTCGTTGATCGTGGTGCAGAGAA
A reverse	Biotin-GGTTGGCAAACTTGAGTGGT
B forward	TTTCACCTTCCATTGCGCAGACTGGAGGAGTTTTCG
B reverse	Digoxigenin-CGGCXCACATTACGCATCGTTCACC
C forward	P-GAGCAGTAGATAATGACTCGCAGAACGAAAAAGGTGAGC
C reverse	TTCCGTCAACCAGGCTTATC
Linker B–C	P-TCTACTGCTCACTCTCCGTATTCTCTTGACTTTCTGCTACTCGCCCACAACCCCACTACCTTTCACCTTC
Linker A–C	P-TGTTTAGTATTGTTCTCTATCCTCTCTGTGGTAGCAGAAAGTCAAGAGAATACGGAGAGT
High-force unzipping	
A forward	ATACTAAACATAATGACTCGTTGATCGTGGTGCAGAGAA
A reverse	GCGTCGGGTGTAATGACTCGGTTGGCAAACTTGAGTGGT
Anchor A forward	P-CACCCGACGCTAATGACTCATTCGCCTGTCTCTGCCTAA
Anchor A reverse	GAAGATCGATAACAGTTCGTCGATGGGTTC
Overhang A (25 b)	P-TGTTTAGTATGAGTCGAAATTCTTACGGCGTGTCAGAGAGGC
Overhang A (123 b)	P-TGTTTAGTATGAGTCGAAATTCTTACGGCGTGTCAGAGAGGCAATGGCTTGACTTGTGGTGGATCACAGTTTGTGAGTAACGGCAAG ATGCGGTAAACACTGTAATGCGAGCTTCATTGACTCGGCTTAAAGTTCCTGGT
B forward	TTTCACCTTCCATTGCGCAGACTGGAGGAGTTTTCG
B reverse	GCGCCTCGTGCATTGCCACATTACGCATCGTTCACC
Anchor B forward	CACGAGGCGCCATTGCATTCGCCTGTCTCTGCCTAA
Anchor B reverse	GAAGATCGATAACAGTTCGTCGATGGGTTC
Overhang B (25 b)	TGACAGGAAACAGCACGCCAGCCTCTCTGACACGCCGTAAGAATTCGATAGGCAGCAGATTCTCTTTTCACCTTC
Overhang B (123 b)	TGACAGGAAACAGCACGCCAACCAGGAACTTTAAGCCGAGTCAATGAAGCTCGCATTACAGTGTTTACCGCATCTTGCCGTTACTCACA AACTGTGATCCACCACAAGTCAAGCCATTGCCTCTCTGACACGCCGTAAGAATTCGATAGGCAGCAGATTCTCTTTTCACCTTC
DNA stretching	
dsDNA (2)	
Forward	CTCCGAATTCTCAGCAAAACGCTATTCACG
Reverse	H_2N-GGTTGGCAAACTTGAGTGGT
dsDNA (4)	
Forward (→A)	P-GAGCAGTAGATAATGACTCGCAGAACGAAAAAGGTGAGC
Reverse (→B)	P-GAAGGTGAACTAATGACTCTTCCGTCAACCAGGCTTATC
Anchor A forward	P-TCTACTGCTCTAATGACTCATTCGCCTGTCTCTGCCTAA
Anchor A reverse	GAAGATCGATAACAGTTCGTCGATGGGTTC
Anchor B forward	P-GTTCACCTTCTAATGACTCATTCGCCTGTCTCTGCCTAA
Anchor B reverse	GAAGATCGATAACAGTTCGTCGATGGGTTC
Confined rotation (3)	
Forward (→A)	P-GAGCAGTAGATAATGACTCGCAGAACGAAAAAGGTGAGC
Reverse (→B)	P-GAAGGTGAACTAATGACTCTTCCGTCAACCAGGCTTATC
Hairpin A	P-TCTACTGCTCATCCGGGCCGTGCAZTGCACGGCCCGGAT
Anchor B forward	P-GTTCACCTTCTAATGACTCATTCGCCTGTCTCTGCCTAA
Anchor B reverse	GAAGATCGATAACAGTTCGTCGATGGGTTC

"Forward" and "reverse" denote PCR primers. "Linker" and "overhang" refer to the part drawn in black in Fig. A. "Anchor" denotes the PCR-primers for the DNA molecules used to attach the DNA constructs to the surface of the sample chamber via dU-biotin or dU-digoxigenin added to the PCR reaction. The numbers in parentheses denote the number of attachable DNA termini provided by the final constructs, P – phosphate, X – dU-digoxigenin, Z – dU-biotin, H_2N – amino group.

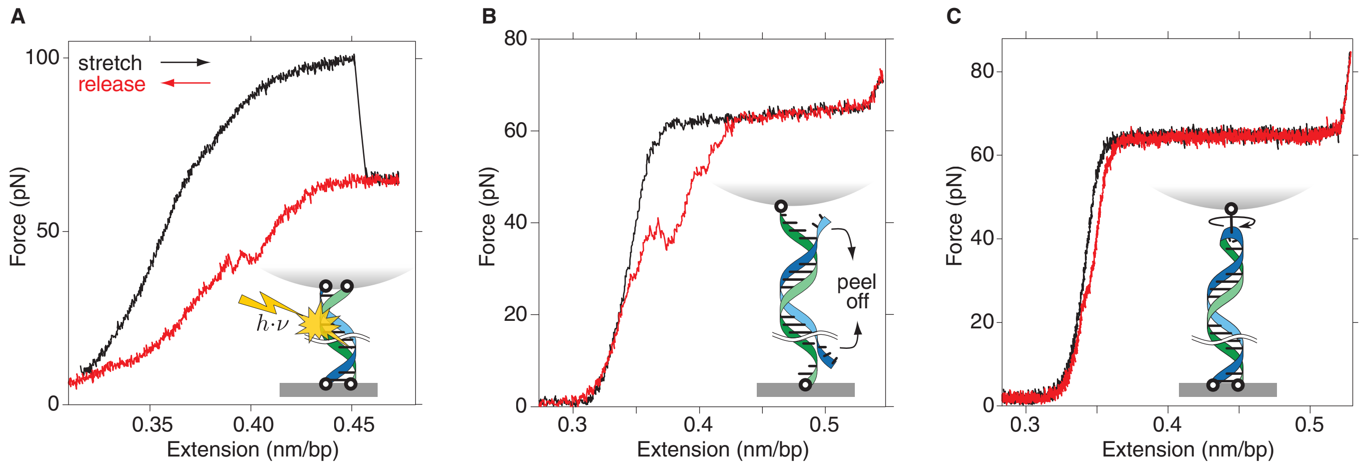

Fig B. Controls of dsDNA force-extension behavior without Redβ. **A**, A 3563 bp-long dsDNA with all termini fixed (inset schematic) was overstretched (black line). A light-induced single-strand break lead to a sudden drop in force and the onset of hysteresis during reannealing upon the release of force (red line). **B**, One strand of a 3133 bp-long dsDNA was fixed on both the microsphere and surface resulting in peeling off of the complementary strand during stretching and subsequent hysteresis. **C**, A 3148 bp-long dsDNA hairpin was overstretched. The hairpin had a single biotin at one end attached to the microsphere with both termini of the other end fixed to the surface. The hairpin was produced by ligation of an interim-biotinylated ssDNA with a PCR-made dsDNA nicked 10 b away from its terminus (Table A). The pulling rate for all experiments was ≈1 μm/s.

≈60 pN in Fig. BA corresponds to the overstretching-plateau force of dsDNA that is only attached on two of its four termini (Fig. BB). Also, in this case, the hysteresis is pronounced since the non-attached termini peeled off. Both the force of the overstretching plateau and the hysteresis are in agreement with the literature [24]. Finally, we stretched a hairpin construct that is closed at one end with rotational freedom and tethered with both termini at the other end (Fig. BC). Since the DNA is free to rotate and strands cannot dissociate, i.e. peel off, the overstretching plateau occurred at ≈60 pN without any annealing hysteresis. These findings are again consistent with the literature [30].

C Circular Dichroism (CD) Measurements Support a Structural Change During Filament Formation

To attribute the larger amplitude of the negative CD band between ≈210–220 nm to a structural change of Redβ, we tested whether the filament CD spectrum can be composed of a weighted sum of the individual spectra recorded for the filament components. Since the concentration of the individual components in the filament sample is only known approximately according to the pipetted concentration (i.e. the concentrations of non-reacted or complexed ssDNA, dsDNA, Redβ, and filament), the filament spectrum might not reflect a structural change but a superposition of the spectra of its components. To rule out this possibility, the filament spectrum can either be matched according to its absorbance (Fig. CA) or the CD (Fig. CB). Figure CA shows the absorbance spectrum recorded with the CD data presented in Fig. 4 of the main text. The best-fit to the filament absorbance by a weighted sum of the individual components ("Fitted sum ABS" curve in Fig. CA) shows a nearly perfect overlap of the absorbance spectrum. We used the weights of the best fit to calculate a weighted sum of the individual CD spectra ("Fitted sum" curve of Fig. 4). This calculated CD spectrum does not account for the increased CD amplitude at ≈220 nm in Fig. 4. Also, for a best fit to the filament CD spectrum (Fig. CB, left panel), the increased CD amplitude at ≈220 nm is not accounted for. In addition, the absorbance spectrum based on the best-fit weights of the filament-CD-spectrum fit showed a significant deviation from the filament spectrum (Fig. CB, arrow right panel). Notably, both best fits invoke negative, thus unrealistic, weights (see the caption of Fig. C).

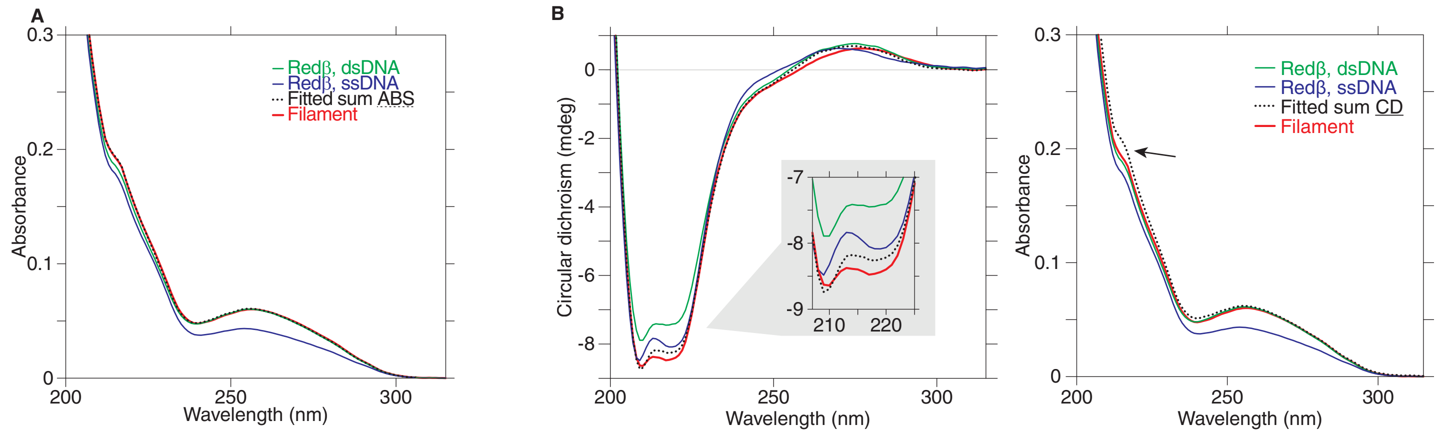

Fig C. Nucleoprotein filament CD spectrum cannot be described as a sum of the individual components. A, Absorbance spectra recorded for Fig. 4 of the main text. The absorbance (ABS) of the filament was best-fitted according to 0.99 ABS(dsDNA+Redβ) - 0.16 ABS(ssDNA) + 0.095 ABS(dsDNA) + 0.052 ABS(Redβ) + 0.008 ABS(ssDNA+Redβ) (dotted line, Fitted sum ABS). **B**, The same CD (left) and absorbance (right) spectra as in Fig. 4 and **A**, respectively, where the filament CD spectrum was best-fit by the reaction components ("Fitted sum CD") as 0.65 CD(dsDNA+Redβ) + 0.023 CD(ssDNA) + 0.22 CD(dsDNA) + 0.44 CD(Redβ) - 0.025 CD(ssDNA+Redβ). The inset shows the region attributed to protein structure. The arrow points at a pronounced difference between the best-fit and filament absorbance spectrum. The same amounts of material were pipetted for all samples (see Methods).

Since the increased CD amplitude cannot be accounted for by any of the components of the annealing reaction, ruling out also potential pipetting errors, the filament CD spectrum implies a structural change of Redβ during the annealing reaction.

Table B. Single-stranded DNAs used for the CD, EMSA, and FCS experiments.

Name	Length (bases)	Sequence
$ssDNA_{PAGE}$	30	`Atto 565-CTTCCACTTTCATCGTCTTTCAGTTCTCTT`
$ssDNA_{FCS}$	30	`Atto 655-ATGCCTCTCACTCGTCATCTTGTTTAGTAT`
$ssDNA_{CD}(1)$	44	`GTAGAGTATAAGCACAGAGAGGATAGAGAACAATGGAGCGGGTGT`
$ssDNA_{CD}(2)$	44	`ACACCCGCTCCATTGTTCTCTATCCTCTCTGTGCTTATACTCTAC`

$ssDNA_{CD}$(1) and (2) were used to generate nucleoprotein filament without single-stranded overhangs for circular dichroism measurements. $ssDNA_{PAGE}$ was used for native polyacrylamide gel electrophoresis. $ssDNA_{FCS}$ was used for fluorescence correlation spectroscopy.

D Redβ Homo-Oligomerizes

Redβ oligomerizes in solution with an oligomerization pattern typical for isodesmic growth [43]. The position of the oligomer bands shown in Fig. 5B was not influenced by ssDNA since the same band pattern of Redβ was observed without ssDNA (Fig. DA). Also, we tested other ssDNA sequences yielding the same results as in Fig. 5B (data not shown). Furthermore, the band pattern was not affected by chemical labeling of Redβ. We observed consistent patterns with (Fig. DA) and without (Fig. DB) chemical labeling of Redβ. In addition, Redβ's ssDNA binding properties were not influenced by the labeling: Our fluorescence correlation spectroscopy measurements of the ssDNA hydrodynamic diameter using chemically labeled Redβ (Fig. 5) and unlabeled Redβ (data not shown) yielded consistent results.

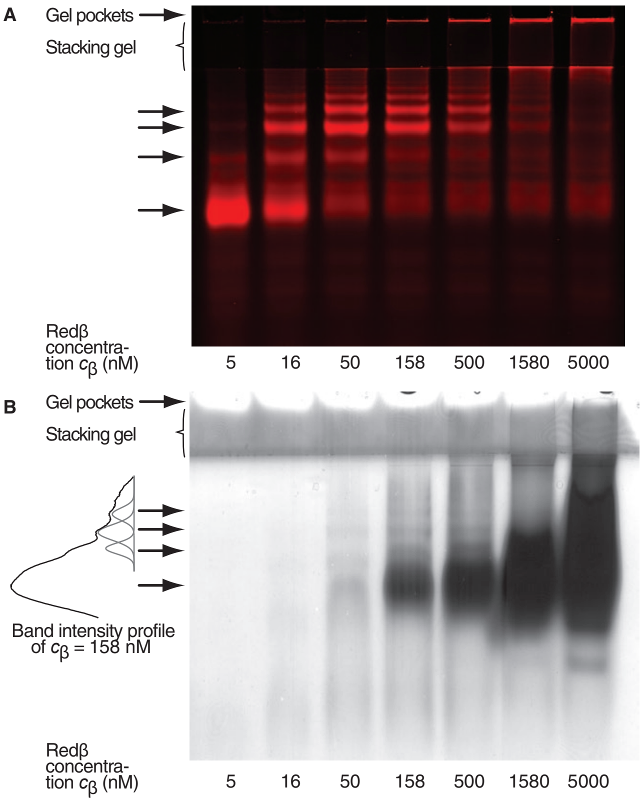

Fig D. Redβ oligomers separated by non-denaturing, polyacrylamide gel electrophoresis. **A**, A constant 5 nM of Alexa 488-labeled Redβ was mixed with unlabeled Redβ. The total Redβ concentration c_β is given below the lanes. No ssDNA was added. Arrows indicate the position of bands in panel **A** and **B**. **B**, Redβ run as in **A**, but without labeled Redβ. The contrast of the silver-stained [91] gel was enhanced to visualize the band pattern. Left: Intensity profile of the c_β =158 nM lane with the discernable bands visualized as Gaussian distributions. We attribute the lowest band appearing at 5000 nM Redβ to an impurity of our sample smaller than 2 % based on the band intensities. Because of several washing steps during the silver staining procedure, hardly any protein is visible in the gel pockets.

E FCS Hydrodynamic Diameter of ssDNA and Force-Extension Measurements Are Consistent with Theory

We calculated the hydrodynamic diameter of freely diffusing ssDNA without Redβ based on the radius of gyration using the ssDNA contour and persistence length [92, 93]. Since these parameters depend on the buffer conditions, we globally determined them from (i) a fit of an extensible freely jointed chain formula to ssDNA force-extension measurements and (ii) a fit of the radius-of-gyration formula to the FCS-measured hydrodynamic diameter. These mechanical parameters of ssDNA are also used to convert the unzipping step size from nanometers to the number of bases that a Redβ monomer binds (Fig. 2C,D).

We calculated the radius of gyration R_g, which is half the hydrodynamic diameter [92], according to [93]

$$R_g^2 = \frac{1}{3} n L_0 L_p - L_p^2 + \frac{2L_p^3}{nL_0} - \frac{2L_p^4}{(nL_0)^2}\left(1 - e^{-nL_0/L_p}\right) \tag{S1}$$

or since $nL_0 \gg L_p$

$$R_g \approx \sqrt{\frac{1}{3} n L_0 L_p}, \tag{S2}$$

where n is the number of bases, L_0 the contour length per base, and L_p the persistence length. To

model our force-extension measurements, we used the extensible freely jointed chain model [24]

$$x = nL_0 \left[\coth\left(\frac{2FL_p}{k_B T}\right) - \frac{k_B T}{2FL_p}\right]\left(1 + \frac{F}{K}\right) + x_0 \tag{S3}$$

or for $FL_p \gg k_B T$

$$x \approx L_0 n (1 + \frac{F}{K}) \tag{S4}$$

where x is the extension, x_0 is an offset accounting for the unknown DNA anchoring point, F is the force, K the elastic modulus, k_B Boltzmann's constant, and T the temperature. To determine the number of bases a Redβ monomer binds, we used Eq. S3 to convert a change in the extension x upon unzipping a nucleoprotein filament (Fig. 2C) to a change in the number of unzipped bases.

To determine the ssDNA mechanical parameters, we used Eq. S3 to fit the force-extension curve of a single-stranded DNA of $n = 3133$ b length and Eq. S1 to fit the FCS-measured hydrodynamic diameter of the $n = 30$ b (Fig. 5C) and $n = 50$ b (data not shown) long oligonucleotides without Redβ. The global best-fit parameters were $L_0 = 0.44 \pm 0.03$ nm/base, $L_p = 0.73 \pm 0.06$ nm, and $K = 0.84 \pm 0.16$ nN. Literature values are in the range of $0.43 \leq L_0 \leq 0.66$ nm/base, $0.75 \leq L_p \leq 3.1$ nm, and $0.53 \leq K \leq 2.2$ nN [24, 51, 92–98]. Our values are consistent with these values. L_0 and L_p are at the lower end of the published ranges, which we attribute to the magnesium ions in our buffer (10 mM $MgCl_2$). Based on our values and using Eq. S1, the calculated hydrodynamic diameter of the 30 b and 50 b ssDNA was 3.6 nm and 4.6 nm, respectively. These values are consistent with the FCS measured diameters of 3.4±0.2 nm (mean±standard deviation, $N = 6$, Fig. 5C) and 4.3±0.1 nm ($N = 3$, data not shown), respectively. In summary, the mechanical parameters for ssDNA are consistent with the literature, and our FCS and optical trapping data.

F Annealing Model

The implementation and fitting of the model was done in MATLAB. We used the structural parameters of Redβ oligomers according to [4]: The Redβ monomer diameter is 4.1 nm in the filament. Eleven monomers assemble into a ring-like structure resembling a split washer—a right-handed helix with a radius of 7.1 nm measured from the center to the mid point of Redβ. 11 Redβ monomers make up one turn with a pitch of 1.4 nm. Because the pitch is smaller than the height of Redβ (3.5 nm), the ring-like structure cannot grow further. Therefore, we modeled a Redβ monomer as a sphere with a diameter of 4.1 nm and oligomers ($n \leq 11$) as n touching spheres on the helix path described above. To calculate the hydrodynamic diameter, we used these shapes as inputs for the freely available software HYDROSUB [42].

To determine the average hydrodynamic diameter, we weighted the diameter of an oligomer species n (with n protomers and up to n fluorophores) by its relative concentration $c_n = [\beta_n]/[\beta]$ according to the isodesmic growth model and a size-dependent brightness factor B_n [99, 100] such that the average hydrodynamic diameter $\overline{d}$ is given by

$$\overline{d} = \frac{\sum_n B_n c_n d_n}{\sum_n B_n d_n}. \tag{S5}$$

Here, d_n is the hydrodynamic diameter of species n according to Fig. 6B. The brightness factor was necessary because the contribution of an individual species to the autocorrelation function in FCS scales with the square of its molecular brightness. Accounting for added unlabeled protein, the brightness factor B_n is given by the second moment of the binomial distribution

$$B_n = ((n+1)\,p)^2 + (n+1)\,p\,(1-p) \tag{S6}$$

where p is the percentage of labeled Redβ. For $c_\beta < 500$ nM, we used fully labeled Redβ ($p = 1$) with a brightness factor of $B_n = n^2$. For $c_\beta \geq 500$ nM, we added unlabeled (c_u) to

Table C. Combinatorial binding model.

N_c	n_{occ}	k	partition	N_p
21	1	1	1	1
11	2	1	01	1
66	2	2	20	1
1	3	1	001	1
1	3	2	110	2
1	3	3	300	1

Example results for the calculation of the weights N_p of individual binding configurations for $M = 30$, $N = 10$. n_{occ} is the number of ligand-sized occupied spaces on the ssDNA. partition contains the numbers and sizes of the bound ligands, $\text{partition} = \{\text{Monomer}, \text{Dimer}, \text{Trimer}, \dots\}$.

labeled Redβ ($c_l = 150\,\text{nM}$) and calculated the labeled percentage according to $p = c_l/(c_l + c_u)$. The addition of unlabeled Redβ lead to a small apparent discontinuity around $c_\beta \approx 500\,\text{nM}$ (red solid line Fig. 5C).

For Redβ-binding to ssDNA, we calculated a statistical weight according to Epstein [45], which reflects the number of configurations, which a certain number of k ligands can assume when binding to a single-stranded DNA of M bases length. With an individual ligand covering N bases, the number of configurations N_c becomes

$$N_c = \frac{(M - kN + k)!}{(M - kN)!\,k!}. \tag{S7}$$

To include the binding of ligands of different oligomeric sizes, their permutations when bound to the ssDNA must be considered, too. We calculated the integer partitions of each configuration and their statistical weight from their elements p_m (see an example in the Table C) as

$$N_p = \frac{(\sum_m p_m)!}{\prod_m (p_m)!}. \tag{S8}$$

Summation of the configurations' weights gave the total statistical weight for a certain number of oligomeric ligands, by which the dissociation constant K_n (Eq. 7) was divided. The division resulted in the effective dissociation constant

$$K_{\text{Epstein}} = K_n/(N_c N_P). \tag{S9}$$

To compare the calculated concentration of Redβ-bound ssDNA θ_{calc} to the measured FCCS percentage of Redβ-bound ssDNA θ, we normalized the measured percentage with the maximum possible experimental value $\theta^{\max}$ (i.e. $\theta_{\text{calc}} = \theta/\theta^{\max}$). The maximum possible cross-correlation $\theta^{\max}$ of our device using a doubly labeled ssDNA substrate (Alexa-488/Atto-647) was measured to be $\theta_{\text{ex}}^{\max} = 0.80 \pm 0.04$ (Wolfgang Staroske, pers. comm.). Based on our model, we compared the FCCS-measured fraction θ of Redβ-bound ssDNA to the calculated fraction

$$\theta = \theta^{\max}\theta_{\text{calc}} = \theta_{\text{fit}}^{\max}\frac{\text{ssDNA}_0 - \text{ssDNA}_{\text{unbound}}}{\text{ssDNA}_0} \tag{S10}$$

where $\theta_{\text{fit}}^{\max}$ is the best-fit maximum cross-correlation, ssDNA_0 is the pipetted concentration of ssDNA and $\text{ssDNA}_{\text{unbound}}$ is the calculated concentration of unbound ssDNA ($n = 0$ in Fig. 6C). The maximum cross-correlation determined by the fit was $\theta_{\text{fit}}^{\max} = 0.92 \pm 0.05$. Also, we fitted a Hill-equation to the cross-correlation data, to determine $\theta^{\max}$ at saturating Redβ concentrations ($[\beta] \to \infty$)

$$\theta = \theta_0 + (\theta_{\text{Hill}}^{\max} - \theta_0) \cdot \frac{[\beta]^x}{K_a^x + [\beta]^x}, \tag{S11}$$

where K_a is the Redβ concentration producing half occupation of ssDNAs, x is the Hill coefficient, and θ_0 accounts for an offset. The best fit resulted in $x = 1.2 \pm 0.1$ (standard error unless noted otherwise), $K_a = (2.1 \pm 0.6) \cdot 10^{-7}$ M, $\theta_0 = 0.02 \pm 0.005$, and a maximum possible cross-correlation of $\theta_{\text{Hill}}^{\max} = 0.92 \pm 0.17$. We used $\theta_{\text{fit}}^{\max}$ to normalize the amplitude of the cross correlation data. Overall, the normalized cross-correlation data were very well described by our model-determined percentage of complexed ssDNA molecules (purple line in Fig. 5C). Enforcing a Hill coefficient $x = 1$ in the fit of Eq. S11 to the FCCS data, resulted in $K_a = (3.9 \pm 0.9) \cdot 10^{-7}$ M and $\theta_{\text{Menten}}^{\max} = 1.29 \pm 0.21$. In this case, the constant K_a is equivalent to the dissociation constant K_d according to Epstein's approach. If we restrict the calculation in Eq. S9 to the binding of a single monomer, an approximate value for K_a can be calculated as $K_a \approx K_{\text{Epstein}} = K_d / N_c N_p = (5.0 \pm 0.2) \cdot 10^{-6}\,\text{M}/21 = (2.4 \pm 0.1) \cdot 10^{-7}$ M, where we used the best-fit value of K_d from Table 1 and $n_{\text{occ}} = 1$ in Table C. The agreement, within error margins, supports a Michaelis-Menten kinetics binding mechanism.

Supplementary References

91. Blum H, Beier H, Gross HJ. Improved silver staining of plant proteins, RNA and DNA in polyacrylamide gels. Electrophoresis. 1987;8(2):93–99.

92. Doose S, Barsch H, Sauer M. Polymer properties of polythymine as revealed by translational diffusion. Biophys J. 2007 Aug;93(4):1224–1234.

93. Sim AYL, Lipfert J, Herschlag D, Doniach S. Salt dependence of the radius of gyration and flexibility of single-stranded DNA in solution probed by small-angle x-ray scattering. Phys Rev E. 2012 Aug;86(2).

94. Mills JB, Vacano E, Hagerman PJ. Flexibility of single-stranded DNA: use of gapped duplex helices to determine the persistence lengths of poly(dT) and poly(dA). J Mol Biol. 1999;285(1):245–257.

95. Murphy MC, Rasnik I, Cheng W, Lohman TM, Ha T. Probing single-stranded DNA conformational flexibility using fluorescence spectroscopy. Biophys J. 2004 Apr;86(4):2530–2537.

96. Laurence TA. Probing structural heterogeneities and fluctuations of nucleic acids and denatured proteins. Proc Natl Acad Sci USA. 2005 Nov;102(48):17348–17353.

97. Johnson DS, Bai L, Smith BY, Patel SS, Wang MD. Single-Molecule Studies Reveal Dynamics of DNA Unwinding by the Ring-Shaped T7 Helicase. Cell. 2007 Jun;129(7):1299–1309.

98. Calderon CP, Chen WH, Lin KJ, Harris NC, Kiang CH. Quantifying DNA melting transitions using single-molecule force spectroscopy. J Phys : Condens Matter. 2009 Jan;21(3):034114.

99. Krouglova T, Vercammen J, Engelborghs Y. Correct diffusion coefficients of proteins in fluorescence correlation spectroscopy. Application to tubulin oligomers induced by Mg^{2+} and Paclitaxel. Biophys J. 2004;87(4):2635–2646.

100. Mozziconacci J, Sandblad L, Wachsmuth M, Brunner D, Karsenti E. Tubulin dimers oligomerize before their incorporation into microtubules. PLoS ONE. 2008;3(11):e3821.
